# The pattern of histone H3 epigenetic posttranslational modifications is regulated by the VRK1 chromatin kinase

**DOI:** 10.1186/s13072-023-00494-7

**Published:** 2023-05-13

**Authors:** Eva Monte-Serrano, Patricia Morejón-García, Ignacio Campillo-Marcos, Aurora Campos-Díaz, Elena Navarro-Carrasco, Pedro A. Lazo

**Affiliations:** 1 Molecular Mechanisms of Cancer Program, Instituto de Biología Molecular y Celular del Cáncer, Consejo Superior de Investigaciones Científicas (CSIC) - Universidad de Salamanca, 37007 Salamanca, Spain; 2 Instituto de Investigación Biomédica de Salamanca (IBSAL), Hospital Universitario de Salamanca, 37007 Salamanca, Spain

**Keywords:** Histone H3, Acetylation, Methylation, Kinase, Chromatin, VRK-IN-1

## Abstract

**Background:**

Dynamic chromatin remodeling is associated with changes in the epigenetic pattern of histone acetylations and methylations required for processes based on dynamic chromatin remodeling and implicated in different nuclear functions. These histone epigenetic modifications need to be coordinated, a role that may be mediated by chromatin kinases such as VRK1, which phosphorylates histones H3 and H2A.

**Methods:**

The effect of VRK1 depletion and VRK1 inhibitor, VRK-IN-1, on the acetylation and methylation of histone H3 in K4, K9 and K27 was determined under different conditions, arrested or proliferating cells, in A549 lung adenocarcinoma and U2OS osteosarcoma cells.

**Results:**

Chromatin organization is determined by the phosphorylation pattern of histones mediated by different types of enzymes. We have studied how the VRK1 chromatin kinase can alter the epigenetic posttranslational modifications of histones by using siRNA, a specific inhibitor of this kinase (VRK-IN-1), and of histone acetyl and methyl transferases, as well as histone deacetylase and demethylase. Loss of VRK1 implicated a switch in the state of H3K9 posttranslational modifications. VRK1 depletion/inhibition causes a loss of H3K9 acetylation and facilitates its methylation. This effect is similar to that of the KAT inhibitor C646, and to KDM inhibitors as iadademstat (ORY-1001) or JMJD2 inhibitor. Alternatively, HDAC inhibitors (selisistat, panobinostat, vorinostat) and KMT inhibitors (tazemetostat, chaetocin) have the opposite effect of VRK1 depletion or inhibition, and cause increase of H3K9ac and a decrease of H3K9me3. VRK1 stably interacts with members of these four enzyme families. However, VRK1 can only play a role on these epigenetic modifications by indirect mechanisms in which these epigenetic enzymes are likely targets to be regulated and coordinated by VRK1.

**Conclusions:**

The chromatin kinase VRK1 regulates the epigenetic patterns of histone H3 acetylation and methylation in lysines 4, 9 and 27. VRK1 is a master regulator of chromatin organization associated with its specific functions, such as transcription or DNA repair.

**Supplementary Information:**

The online version contains supplementary material available at 10.1186/s13072-023-00494-7.

## Introduction

Chromatin remodeling is a basic process required for the adaptation of specific genomic regions to different biological processes, such as gene transcription, gene silencing, replication, chromosome condensation in mitosis, recombination, and DNA damage responses [1]. All these processes require a dynamic adaptation of chromatin to different functional requirements, as these processes progress, and may also differ among different chromosomal regions such as euchromatin or heterochromatin. The dynamic reorganization of chromatin is associated with epigenetic modification of DNA and histones, gene expression or silencing. Furthermore, chromatin remodeling also underlies cell fate and identity [2]

Some epigenetic modifications of histones are associated with the regulation of genes and its methylation with transcriptional silencing [3, 4]. The H3K9ac and H3K27ac marks are mainly surrounding promoters and enhancers [5, 6], occur in gene transcription, and their methylation is also associated with gene silencing [6], and affects cell identity [7]. The two alternative epigenetic modifications in these two H3 lysine residues require the coordination of the different epigenetic enzymes implicated that add, or remove, acetyl and methyl groups. Furthermore, the methylation of H3 in K4 is associated with gene transcription [6, 8].

The different and alternative epigenetic modifications of histones, such as acetylation or methylation in the same lysine residue, requires writers and erasers that belong to different enzyme families, which need to be coordinated based on their particular functional role. These epigenetic modifications control the degree of chromatin compaction or relaxation as well as the recruitment of proteins associated with specific functions [9, 10]. The coordination of the epigenetic enzymes implicated is likely to be mediated by kinases, which indirectly control the pattern of these histone modifications. All of these epigenetic enzymes are coordinated or regulated by phosphorylation in different specific enzymes, which may differ depending on their function.

Among the candidates to play a regulatory role of epigenetic modifications is VRK1, a chromatin kinase that appeared late in evolution in pluricellular eukaryotes. It is not present in yeasts, and in *Drosophila melanogaster* this gene is unique and known as NHK-1 (nucleosomal kinase-1) [11]. The VRK1 kinase domain is distantly related to casein kinases and insensitive to inhibitors targeting different kinase families [12–14]. The VRK1 protein has characteristics that make it distinct and candidate for development of highly specific inhibitors [13], one of which has been recently developed [15, 16]. VRK1 has been associated with the control of cell proliferation [17] and mitosis [18, 19], and is expressed at high levels in proliferating and tumor cells [17, 20], which is an indicator of a poorer prognosis in many tumor types [21–25]. VRK1 is also necessary to maintain genomic stability and its loss facilitates DNA damage by a defective DNA damage response [20, 26].

Among VRK1 direct nucleosomal phosphorylation targets are histone H3 in Thr3 [19, 27] and Ser10 [28], and H2A in Thr120 [11]. H4K16 acetylation controls chromatin organization and protein interactions [29]. The phosphorylation of histone H3 in Thr3 is required for phosphorylating and recruiting KAT5/Tip60 to chromatin [30, 31]. VRK1 phosphorylates and activates Tip60/KAT5 and regulates the acetylation of H4K16 [30, 31]. In DNA damage responses (DDR), VRK1 also phosphorylates H2AX in Ser139 (γH2AX) [32]. Furthermore, VRK1 also directly phosphorylates several transcription factors such as p53 [33, 34], c-Jun [35], ATF2 [36] and CREB [37], and all of phosphorylation are lost by VRK1 depletion. Furthermore, VRK1 inhibition also impairs H3Thr3 phosphorylation in a dose-dependent manner [38]. These phosphorylations, mediated by VRK1, are functionally associated with gene transcription, cell cycle progression, mitosis, and DNA damage responses (DDR) [26], and it is of note that all these processes require a dynamic and sequential remodeling of chromatin [20, 39]. VRK1 impairs G0 exit, because is required for *CCND1* gene (cyclin D1) expression [17], and G2/M in cell cycle progression [19].

Because of the roles that VRK1 has on the regulation of several chromatin-associated proteins, we have studied whether VRK1 is indeed affecting the epigenetic pattern of histone posttranslational modifications (PTMs), mainly acetylation and methylation, in specific histone lysine residues. For this aim we used depletion of VRK1 with siRNA, or its inhibition with VRK-IN-1, to determine how histone H3 epigenetic PTM patterns are modified. Modifications of the histone epigenetic patterns can have pathological and therapeutic implications because of their role in DDR and other functions requiring a dynamic chromatin remodeling in tumor biology.

## Results

### VRK1 depletion alters the epigenetic pattern of histone H3K9 acetylation and methylation

**Fig. 1:**
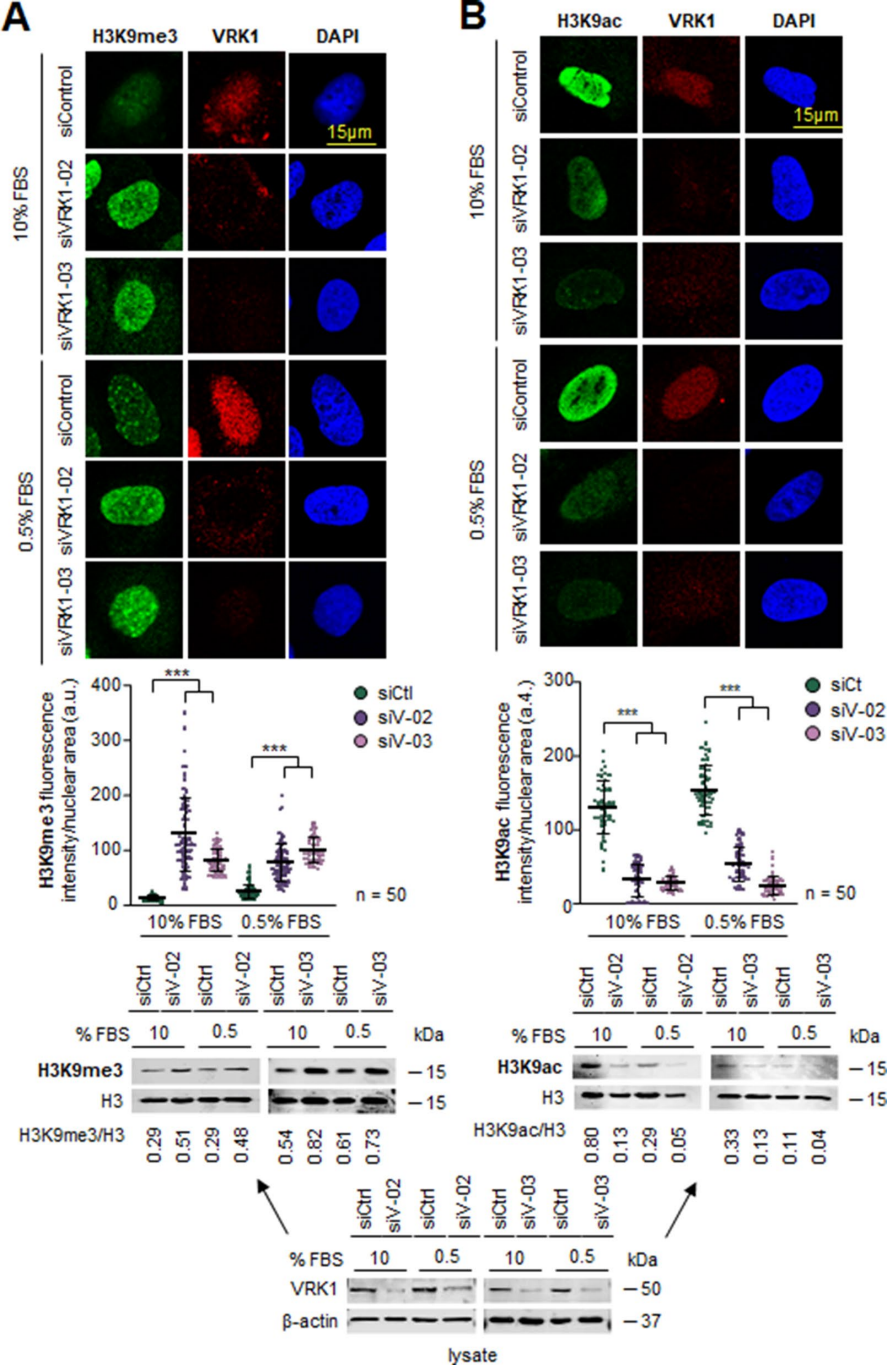
Effect of VRK1 depletion on epigenetic modifications of H3K9 in the presence or absence of serum in A549 cells. The field figure is shown in Additional file 1: Fig. S1. **A** Effect of VRK1 depletion on histone H3K9 methylation in A549 cells. **B** Effect of VRK1 depletion on histone H3K9 acetylation in A549 cells. The quantification of H3K9 methylation (left) and acetylation (right) from 50 cells are shown below. The immunoblots of the cell extracts from the experiment are shown at the bottom. The lysate was divided in two for loading in gels to determine each of the H3K9 modifications. ***p < 0.001. siCt, siControl; siV-02, siVRK1-02; siV-03, siVRK1-03. The same lysate was used for both determinations

The acetylation and methylation of H3K9 is associated with gene transcription and repression, and they are mainly located in the gene body. The effect of VRK1 depletion, using two different siRNA on epigenetic modifications of histone H3K9 was determined in two different cell lines, A549 lung adenocarcinoma and U2OS osteosarcoma, under two conditions, in the presence and absence of serum. VRK1 depletion caused an increase in H3K9 methylation (Fig. 1a; Additional file 1: Fig. S1A) and a decrease in H3K9 acetylation (Fig. 1b; Additional file 1: Fig. S1B) in both A549 (Fig. 1) and U20S (Additional file 2: Fig. S2) cells cell lines independently of serum, which is consistent with a reduction in transcription. H3K9 methylation is associated with repressive chromatin [40].

### VRK1 depletion alters the epigenetic pattern of histone H3K27 acetylation and methylation

**Fig. 2:**
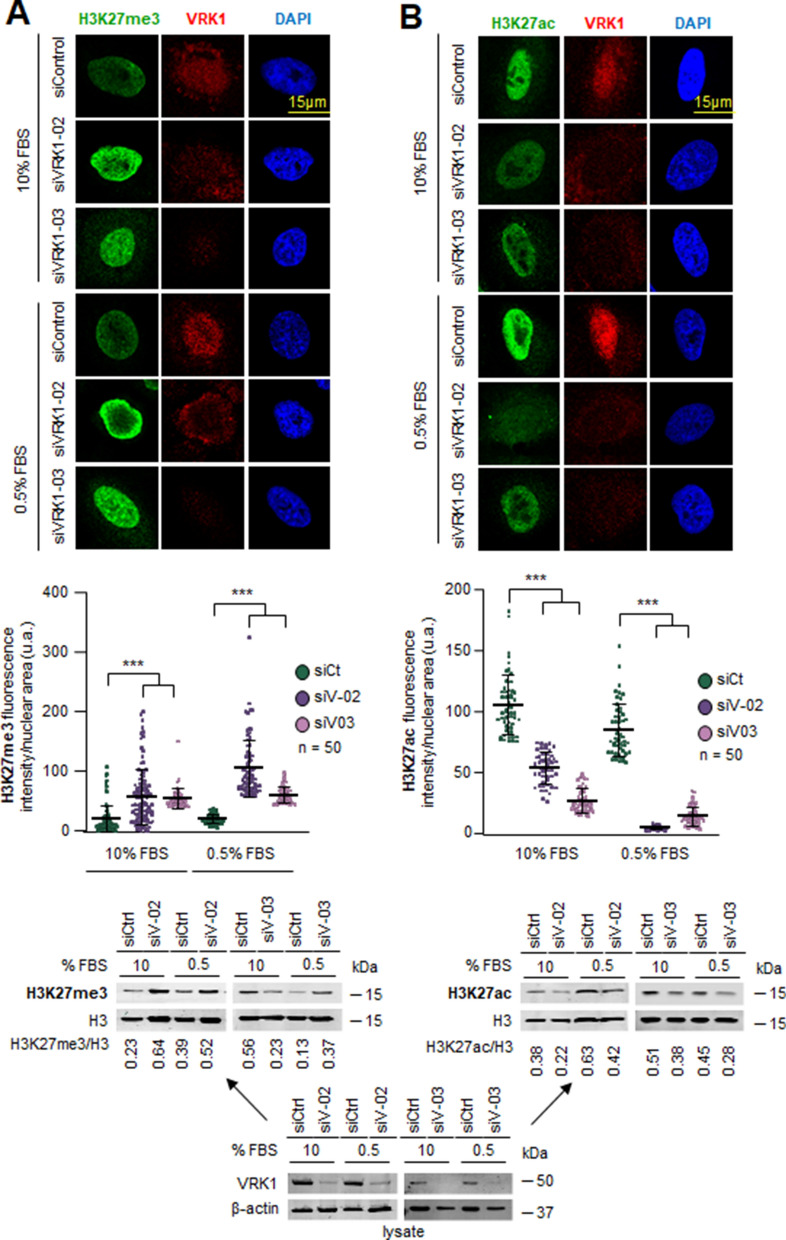
Effect of VRK1 depletion of epigenetic modifications of H3K27 in the presence or absence of serum in A549 lung adenocarcinoma cells. The field figure is shown in Additional file 3: Fig. S3. **A** Effect of VRK1 depletion on histone H3K27 methylation in A549 cells. **B** Effect of VRK1 depletion on histone H3K9 acetylation in A549 cells. The quantification of H3K27 methylation (left) and acetylation (right) from 50 cells is shown below. The immunoblots of the cell extracts from the experiment are shown at the bottom. The lysate was divided in two for loading in gels to determine each of the H3K27 modifications. ***p < 0.001. siCt, siControl; siV-02, siVRK1-02; siV-03, siVRK1-03

H3K27 acetylation is associated with active enhancers [5, 41, 42] and its methylation with repression of transcription [42–44]. The effect of VRK1 depletion, using two different siRNA on epigenetic modifications of histone H3K27 was determined in two different cell lines, A549 and U2OS. VRK1 depletion caused an increase in H3K27 methylation (Fig. 2a; Additional file 3: Fig. S3A) and a decrease in H3K27 acetylation (Fig. 2b; Additional file 3: Fig. S3B) in both A549 (Fig. 2) and U2OS cells (Additional file 4: Fig. S4) independently of the presence or absence of serum.

### The VRK-IN-1 inhibitor alters the epigenetic pattern of histone H3K4, H3K9 and H3K27 modifications

**Fig. 3:**
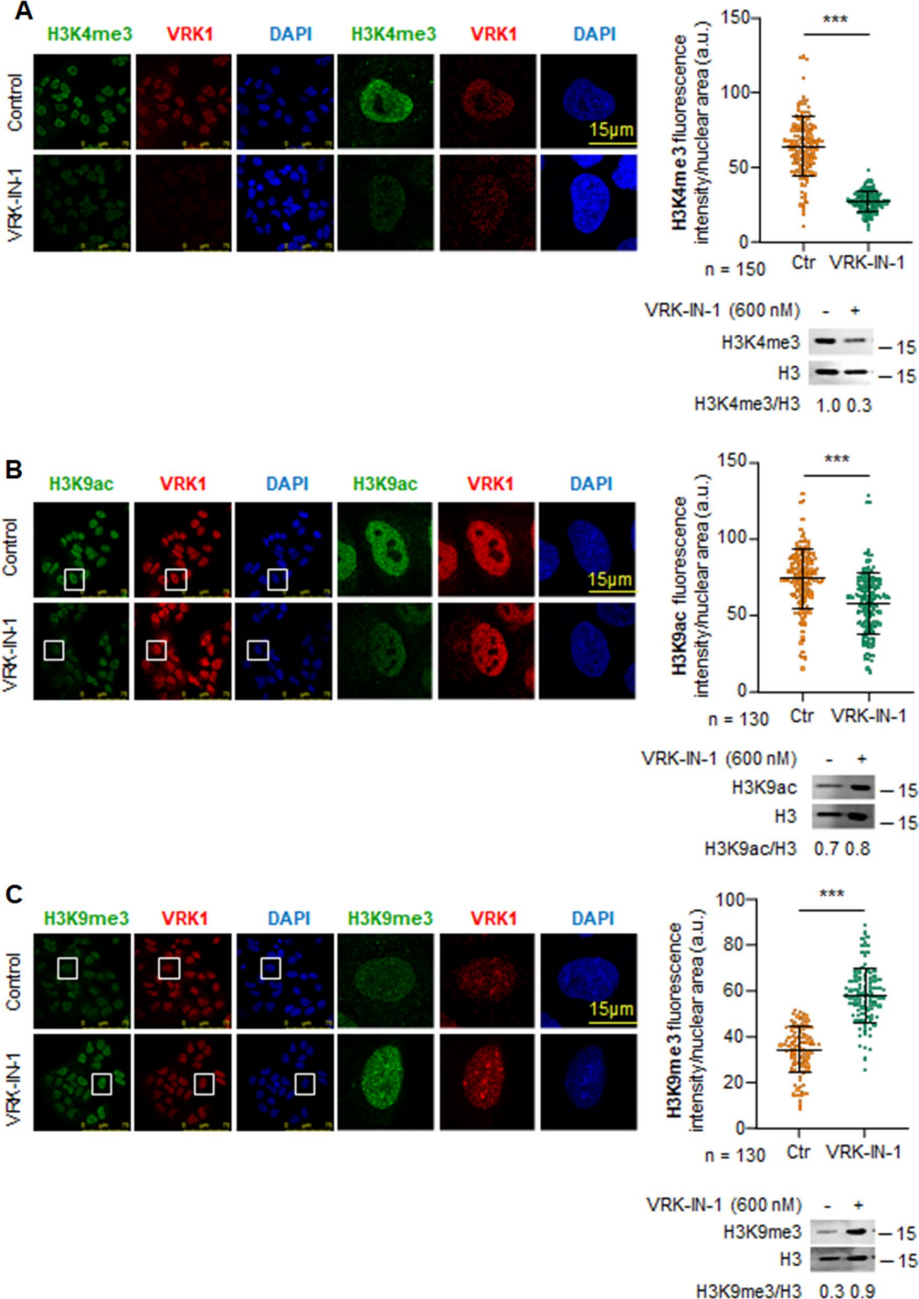
Effect of VRK1 depletion or inhibition with VRK1-IN-1 on epigenetic modifications of H3K4 and H3K9 in A549 cells the presence or absence of serum. **A** Effect of VRK-IN-1 on histone H3K4m3 levels. **B** Effect of VRK-IN-1 on histone H3K9ac levels. **C** Effect of VRK-IN-1 on histone H3K9m3 levels. Ctr: control. ***p < 0.001

A novel and specific inhibitor of VRK1 (VRK-IN-1), based on a pyridine structure, has been recently developed [15, 16]. Therefore, we tested the effect of VRK-IN-1 inhibitor in A549 and U2OS cells either in the presence and absence of serum to determine whether it can mimic the effect of VRK1 depletion on H3 posttranslational epigenetic modifications. Under both conditions, VRK1 inhibition in A549 cells resulted in a loss of H3K4me3 (Fig. 3a), a mark associated with promoters and gene transcription [45, 46]. Inhibition of VRK1 resulted in a loss of H3K9 acetylation (Fig. 3b) and a gain of H3K9me3 (Fig. 3c). Regarding H3K27, a similar but less pronounced result was obtained by inhibition with VRK-IN-1, a loss of H3K27 acetylation (Additional file 5: Fig. S5) and a gain of H3K27me3 (Additional file 5: Fig. S5). Similar results were obtained in U2OS cells treated with VRK-IN-1 for H4K16ac (Additional file 6: Fig. S6A), H3K4me3 (Additional file 6: Fig. S6B), H3K9ac (Additional file 7: Fig. S7A), H3K9me3 (Additional file 7: Fig. S7B), H3K27ac (Additional file 8: Fig. S8A) and H3K27me3 (Additional file 8: Fig. S8B). The effect of VRK1 inhibition or depletion on histone posttranslational modifications (PTMs) has to be indirect, and mediated by the enzymes performing these specific modifications.

### Inhibitors of epigenetic enzymes mimic the effects of either VRK1 inhibition or depletion on H3K9 acetylation and methylation

**Fig. 4:**
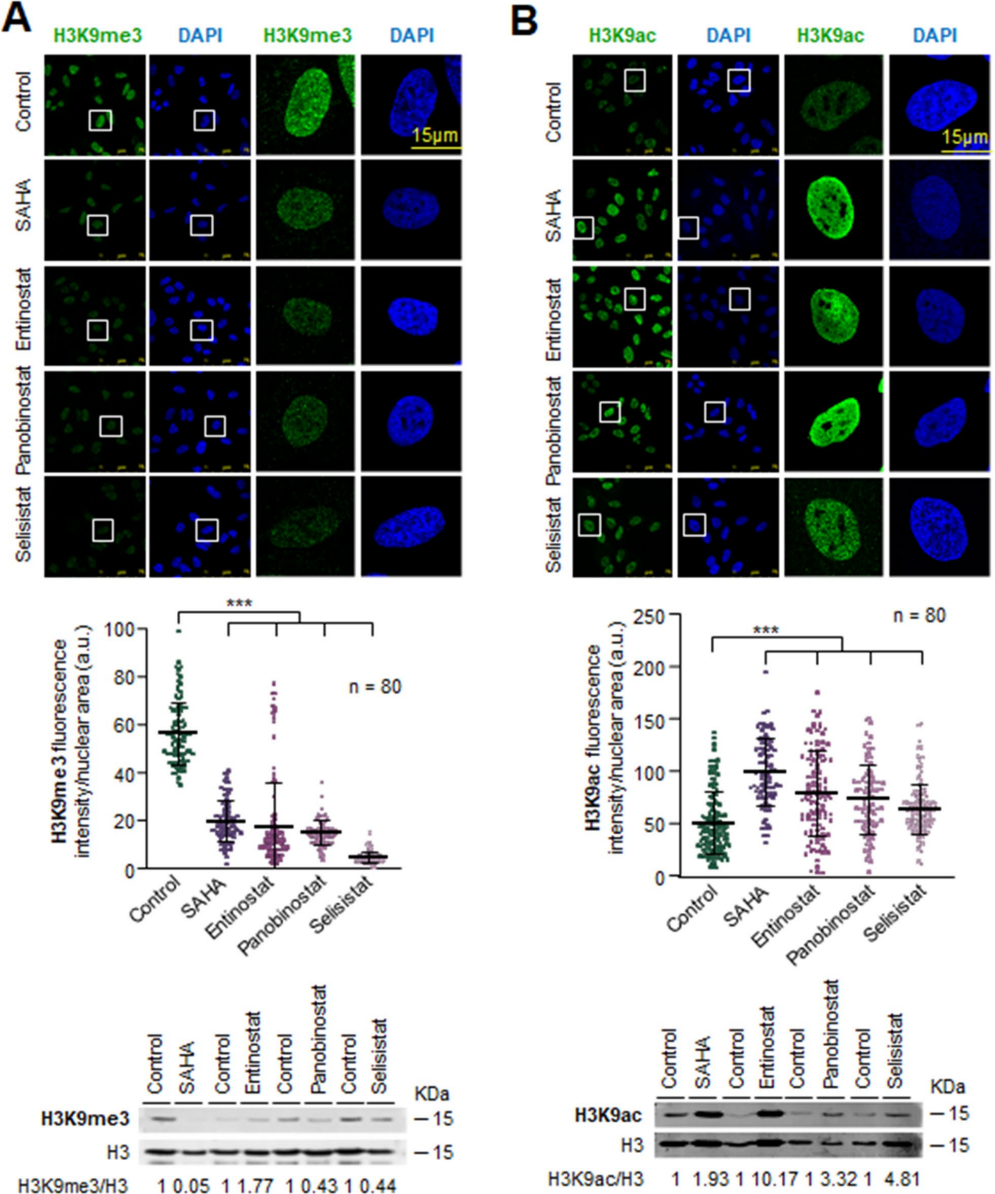
Effect of HDAC inhibitors on histone H3K9 epigenetic modifications in serum-deprived A549 cells. **A** Effect of HDAC inhibitors on H3K9m3 levels. **B** Effect of HDAC inhibitors on H3K9ac levels. The middle panel shows the quantification of the fluorescence. The immunoblots of the two modifications are shown at the bottom. ***p < 0.001

**Fig. 5:**
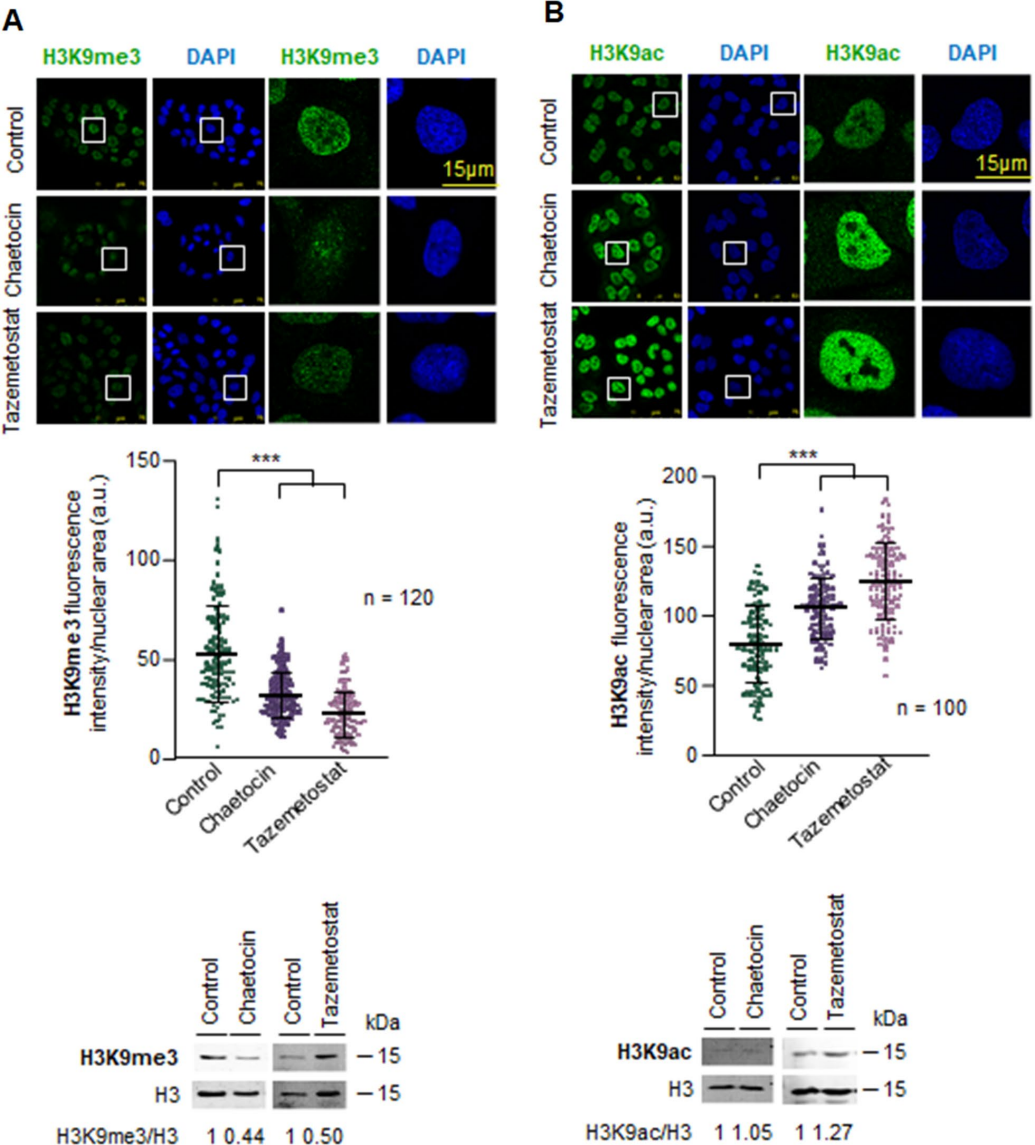
Effect of KMT inhibitors, tazemetostat and chaetocin, on histone H3K9 epigenetic modifications in serum-deprived A549 cells. **A** Effect of KMT inhibitors on H3K9m3 levels. **B** Effect of KMT inhibitors on H3K9ac levels. The middle panel shows the quantification of the fluorescence. The immunoblots of the two modifications are shown at the bottom. ***p < 0.001

The effect of VRK1 depletion, or theVRK-IN-1 inhibitor, on histones PTMs is indirect and has to me mediated by the epigenetic enzymes that add or remove acetyl or methyl marks. Therefore, we determined whether the effects on H3K9 epigenetic posttranslational modifications by the use of inhibitors targeting the four types of enzymes directly involved in these histone PTMs and that might mimic the effect of VRK1 loss. Inhibition of HDAC1 with vorinostat, entinostat, panobinostat or selisistat resulted in an accumulation of acetylation and a consequent reduction of methylation in A549 cells (Fig. 4) and U2OS cells (Additional file 9: Fig. S9). KMT1 inhibition with either chaetocin or tazemetostat facilitated H3K9 acetylation and inhibited H3K9 methylation in both A549 (Fig. 5) and U2OS cell lines (Additional file 10: Fig. S10).

Alternatively, the inhibition of KAT with the C646 inhibitor of p300/CBP acetyl transferase caused a reduction of H3K9ac and an increase in H3K9me3 levels in both cell lines [47] (Additional file 11: Fig. S11). Alternatively, inhibition of KDM1A with ORY-1001 (iadademstat) [48], or JMJD2i caused an accumulation of H3K9 methylation and a loss of H3K9 acetylation (Additional file 12: Fig. S12) in both A549 and U2OS cell lines.

### VRK1 stably interacts with lysine acetyl transferases (KAT) and histone deacetylases (HDAC)

**Fig. 6:**
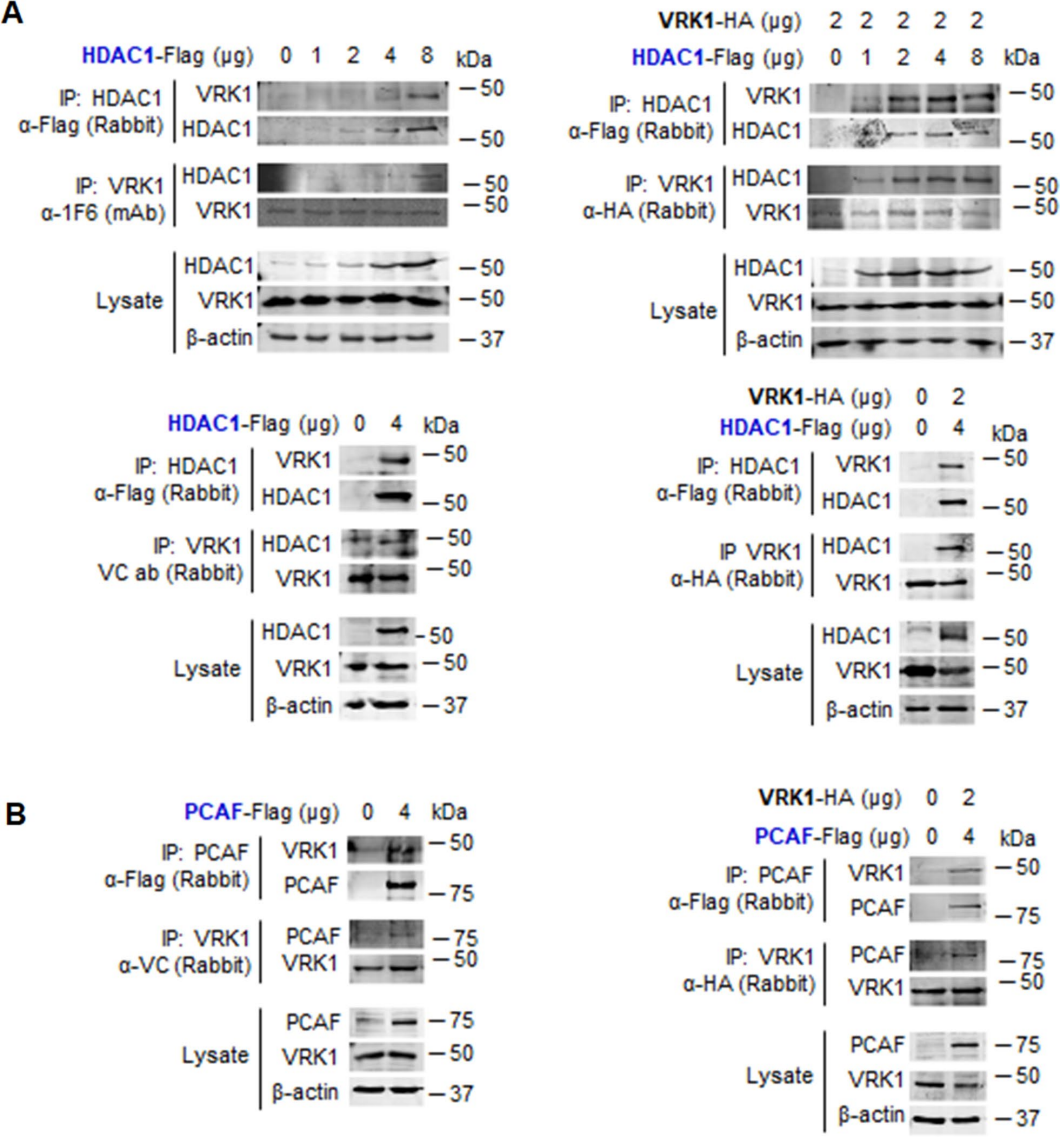
Interaction of HDAC and KAT with VRK1. **A** Left: reciprocal interaction of endogenous VRK1 with increasing amount of transfected HDAC-Flag. Right: dose-dependent interaction between VRK1-HA and increasing amounts of HDAC1-Flag. **B** Left: interaction of endogenous VRK1 with transfected PCAF-Flag. Right: reciprocal interaction of transfected VRK1-HA and PCAF-Flag. Transfections were performed in HEK293T cells

The role of VRK1 in the modulation of epigenetic histone modifications is likely to be due to its interaction with members of these protein families. It is known that VRK1 interacts with the Tip60/KAT5 and regulates its translocation from nucleoplasm to chromatin and its activity [30, 31]. Therefore, it is likely that VRK1 might also form complexes with other members of these epigenetic enzyme families. To test this possibility, we studied the interaction of endogenous VRK1 with tagged HDAC1 transfected in HEK293T cells. The VRK1–HDAC1 interaction was detected in reciprocal immunoprecipitations (Fig. 6a, left), but in the case of immunoprecipitation with an antibody targeting the endogenous VRK1 protein, the detection is weaker because of the interference between its binding to either the antibody or the HDAC1 protein. Therefore, the direct interaction was confirmed by using both tagged proteins (Fig. 6a, right).

Next, we performed a similar experiment to detect the interaction between endogenous VRK1 and the PCAF (KAT2B) acetyl transferase. PCAF interacted with tagged and endogenous VRK1 (Fig. 6b, left). When both PCAF and VRK1 were tagged, both proteins were detected in reciprocal immunoprecipitations (Fig. 6b, right). The direct PCAF–VRK1 interaction was further confirmed in a dose-dependent pull-down in vitro experiments using GST-PCAF and His-VRK1 that were bacterially expressed and purified proteins (Additional file 13: Fig. S13A). This direct interaction was confirmed by transfecting tagged PCAF-flag, which immunoprecipitated the endogenous VRK1 protein in a dose-dependent manner (Additional file 13: Fig. S13B).

### VRK1 stably interacts with histone methylases (KMT) and histone demethylases (KDM)

**Fig. 7:**
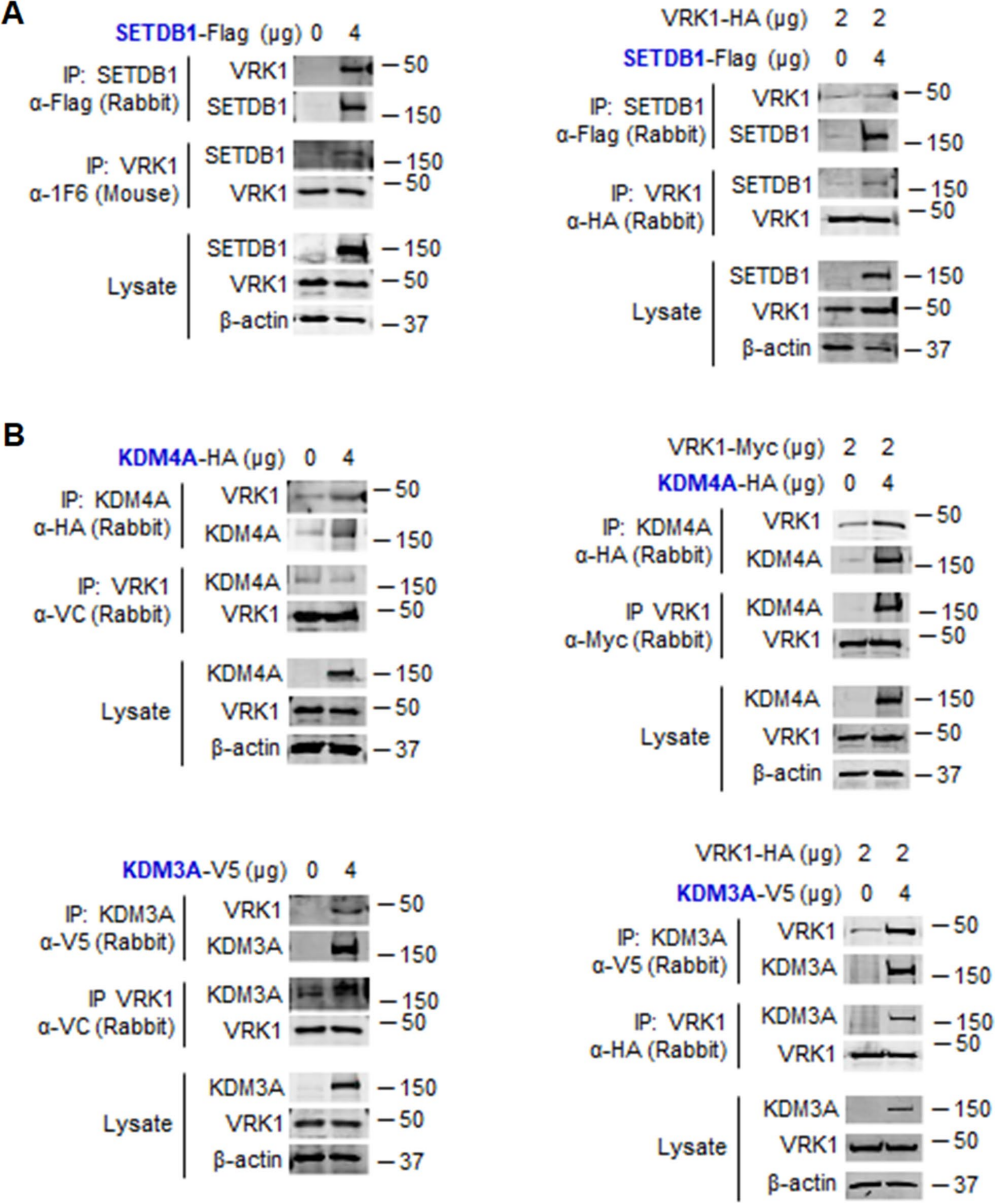
Interaction of SETDB1 (KMT1E) and KDM with VRK1. **A** Left: reciprocal interaction of SETDB1 with endogenous VRK1. Right: reciprocal interaction of SETDB1-Flag (KMT1E) with transfected VRK1-HA. **B** Left: reciprocal interaction of KDM4A (JMJD2A)-HA (top) or KDM3A (JMJD1A)-V5 (bottom) with endogenous VRK1. Right: reciprocal interaction of JMJD2A-HA (top) or KDM3A-V5 (bottom) with transfected VRK1-HA. Transfections were performed in HEK293T cells

Next, we performed similar experiments to detect direct interaction of VRK1 with KMT and KDM proteins. First the interaction with the SETDB1 (KMT1E) enzyme was determined. SETDB1 interacted with endogenous VRK1 (Fig. 7a, left) or transfected VRK1-HA (Fig. 7b, right) in reciprocal immunoprecipitations.

Also, similar experiments were performed with two different KDMs, JMJD2A (KDM4A) and KDM3A. These two KDMs interacted with endogenous (Fig. 7b, left) or transfected VRK1-HA (Fig. 7b, right) in reciprocal immunoprecipitations. VRK1 forms stable protein complexes with SETDB1 (KMT1E), JMJD2A (KDM4A) and JMJD1A (KDM3A).

## Discussion

**Fig. 8:**
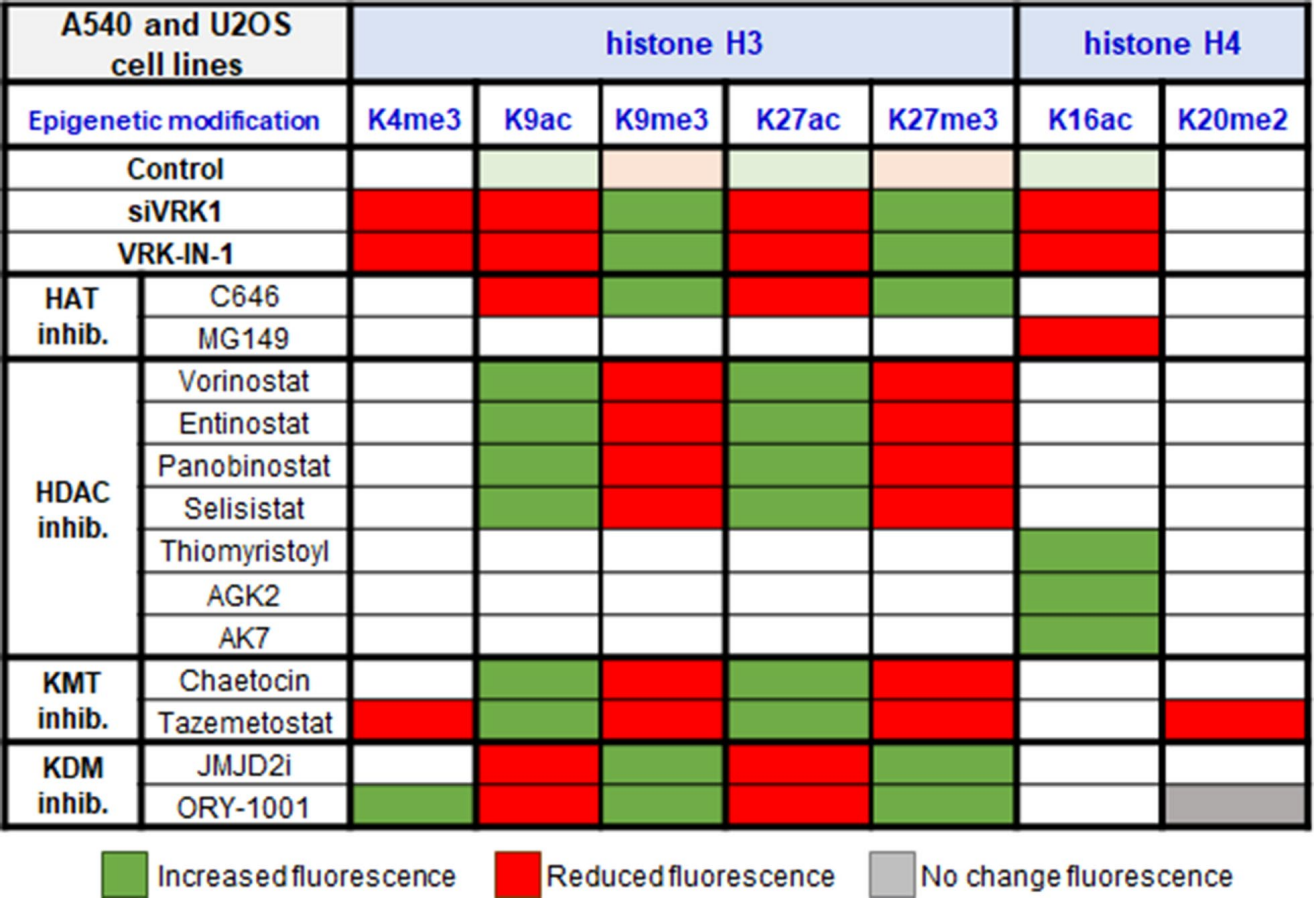
Alteration of the balance and pattern of histone epigenetic posttranslational modifications caused by drugs targeting different enzymes in A549 and U2OS cells

The different functional states of chromatin organization are associated with different combinations of epigenetic posttranslational modifications of histones, which are mediated by enzymes belonging to different protein families, each with multiple members. Among them are kinases, KATs, HDACs, KMTs and KDMs. Furthermore, as biological processes such as transcription, replication, recombination or DDR progression, there is a dynamic change in the local pattern of PTMs, which can vary depending on the stage of the biological process, and can determine its functional role. This requires the coordination of different enzymes among which are KATs, HDACs, KMTs, and KDMs, which introduce different PTMs that are involved in these dynamic processes, in which kinases can play a relevant coordinating role. In this context, the switch in histone epigenetic PTMs caused by VRK1 depletion, or the specific inhibition of its kinase activity (Fig. 8), indicates that there is a significant alteration in chromatin organization, which can be exploited for therapeutic applications. This blockade in the dynamic regulation of chromatin mediated, or coordinated, by VRK1 impairs processes associated with preventing activation of transcription factors such as c-Jun [35], CREB [37], ATF2 [36], p53 [33, 49], or proteins implicated in DDR such as H2AX [32], NBS1 [50], or 53BP1 in non-homologous-end joining (NHEJ) [51], which is recruited H4K20me2 [52, 53], a specific histone modifications that is also involved in nucleotide excision repair (NER) [54].

VRK1 depletion or inhibition caused a reduction of gene expression in different cell lines, which is consistent with the modification in the PTMs patterns of H3K9 and H3K27 associated with these treatments. The effect of VRK1 depletion on H3K9 has also been detected in human glioblastoma cell lines [55]. Cells blocked in a histone H3K9 and H3K27 methylated state are unlikely to be able to permit a correct DNA damage response, and thus sensitize cells to treatments with these drugs. This configuration will also impair gene transcription, which is an effect of VRK1 depletion or inhibition independent of cell type [21, 24, 35, 36, 56]. Furthermore, the transcriptional H3K4me3 epigenetic mark [57] is also impaired by either VRK1 inhibition or depletion. These data are consistent with the known role of VRK1 in the regulation of cell proliferation [21, 58, 59]. Inhibition of KDM impairs the acetylation of methylated lysine residues. In pancreatic cancer cells the expression of PD-L1 is very high [60], and in these tumors the loss of H3K4me3 reduces the expression of PD1-L1 [60]. In this context, targeting VRK1 might be a potentially useful synthetic lethality strategy in combination with immunotherapy approaches targeting the PD1/PD1-L1 axis, which need to be explored. Alteration in epigenetic patterns have also been associated with allergic diseases [61], in which the role of VRK1 has not been studied.

Different drugs targeting enzymes that introduce histone PTMs such as KATs, HDACs, KMTs or KDMs are currently in use as therapeutic drugs. Among them is iadademstat (ORY-1001) targeting KDM1A/LSD1 and used in AML [48, 62], Ewing sarcoma [63], and triple negative breast cancer [64], but their effectiveness can be improved if combinatorial strategies with other drugs are identified. Specific pharmacological targeting of VRK1 can mimic the combined effects of drugs targeting specific epigenetic enzymes. Therefore, the potential use of a kinase inhibitor combined with specific inhibitors of histone epigenetic enzymes can permit a reduction in their doses.

A potential synthetic lethality strategy can be based on the simultaneous targeting of KMT inhibition with tazemetostat that impairs H4K20me2 required for the recruitment of 53BP1 in response to DNA damage in sarcomas [65]. Its combination with a VRK1 inhibitor targeting different pathways, such as proliferation signals, as well as DNA responses such as those mediated by p53, or those associated with different and early steps in DDR such as the formation of γH2AX foci in the response to doxorubicin or ionizing radiation [51, 66] regulated by VRK1 or olaparib, a PARP inhibitor [67]. Furthermore, inhibition of histone H4 acetylation impairs DDR, which by itself may not be enough [68], but can cooperate with VRK1 inhibition in the elimination of tumor cells [30, 31].

The consequence of the drug combination can have two main effects, a reduction of their individual dose and thus of toxic side effects, as well as a reduction of the selective pressure to develop resistance to a specific drug. Drug combinations also permit the targeting of difference pathways. In the case of the VRK-IN-1 inhibitor, it not only targets VRK1, but also the cytosolic and membrane-bound VRK2 [16], which can impair mitogenic signaling [69, 70], and thus can simultaneously block two different tumorigenic pathways. Targeting VRK1 has been shown to be a suitable target in glioblastomas in which the VRK2 gene is silenced [71, 72]. A strategy that is likely to be applicable to other tumors, and even improved if combined with other drugs targeting other epigenetic enzymes from different families.

An example of the relation between VRK1 and epigenetic enzymes is represented by its interaction with Tip60 and HDAC1, two enzymes that regulate the acetylation of histone H4.

**Fig. 9:**
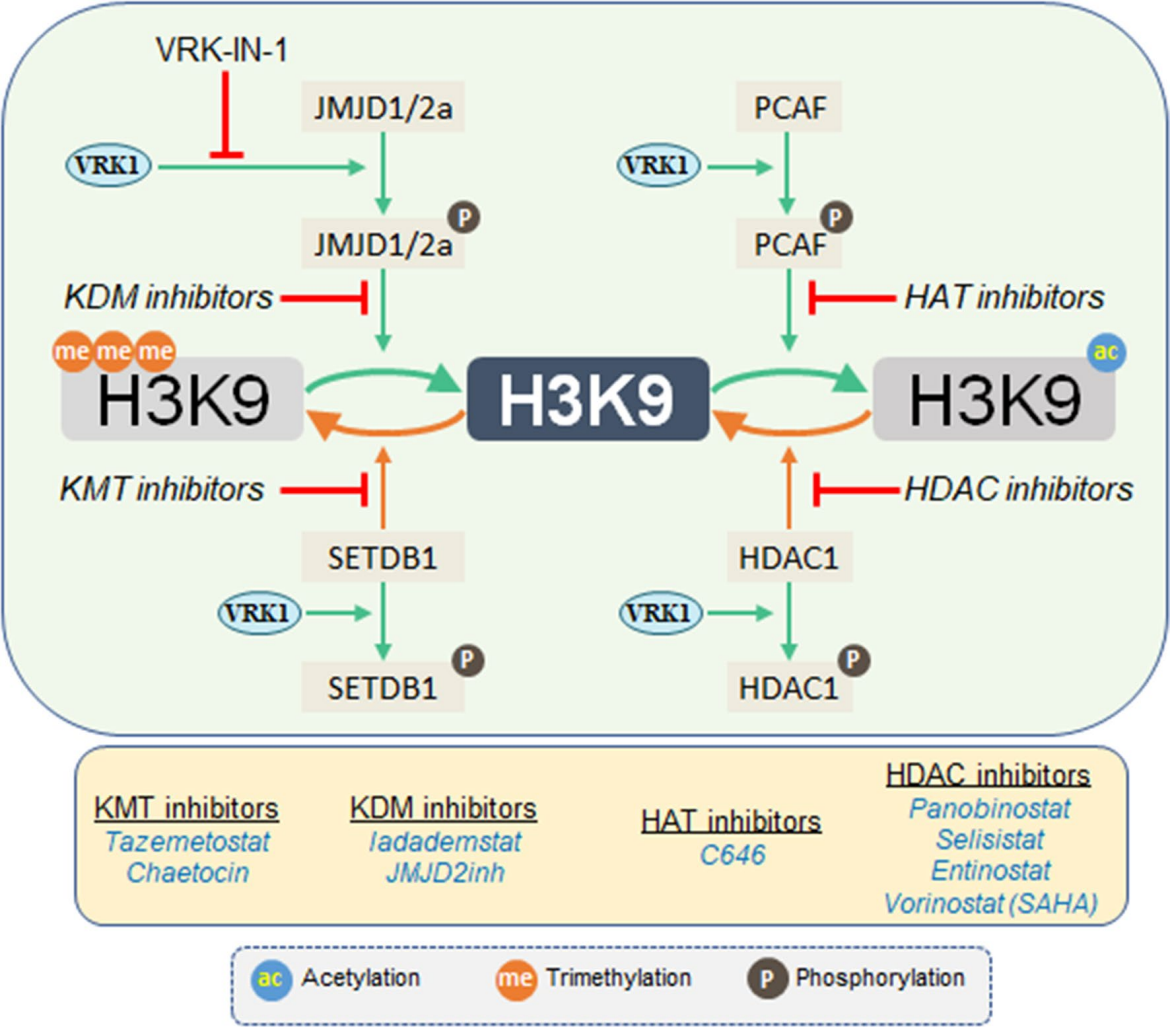
Diagram illustrating the complex regulation of the epigenetic modifications of histone H3K9, and its manipulation by the use of different types of inhibitors

Previously, it has been shown that VRK1 specifically phosphorylates Tip60/KAT5 facilitating its translocation to chromatin and activating the acetylation of H4K16 in the response to DNA damage [30, 31]. The reversal of this acetylation requires histone deacetylases (HDACs). In this context, it has been shown that the deacetylase SIRT2, by interacting with VRK1, inhibits its kinase activity [73], and facilitates the de-acetylation mediated by SIRT2, and thus preventing the re-acetylation of the target protein. HDAC inhibition also facilitates the impairment of DDR by preventing the dynamic remodeling of chromatin associated with the progression of DNA damage responses (DDR) [74]. In this context, the crosstalk in the regulation of H4K16 acetylation by different enzymes supports its potential for pharmacological intervention. The modification in the pattern of H3 and H4 histone acetylation, resulting from by the loss of VRK1, is similar to that caused by KAT inhibitors. Both VRK1 or KAT inhibition result in a defective DDR [31, 75, 76]. Alternatively, VRK1 depletion facilitates histone methylation, a similar effect to that of KDM inhibitors. The maintenance of a histone methylated state also impairs progression of DDR [77, 78]. Thus, the patterns of different combinations of histone epigenetic acetylations and methylations determine the functional situation at specific chromatin locations on genes and chromosomes, which need to be identified in order to associate specific PTM patterns and functions. The use of inhibitors targeting these epigenetics marks blocks chromatin in a specific configuration that is unable to remodel in order to perform specific functions. The complex regulation of the coordination of the epigenetic PTMs on an individual histone is exemplified for H3K9 (Fig. 9). There are four different enzyme activities requiring their coordination, among which VRK1 is a candidate, but other kinases can also be implicated, but need to be identified. Therefore, the formation of stable complexes between VRK1 and several different epigenetic types of enzymes suggests that it is likely that they participate in the regulation of different PTMs, which need to be individually characterized in a manner similar to that of the VRK1-KAT5/Tip60 and HDAC1 [30, 31, 38], in order to determine their role and therapeutic potential.

We concluded that the pattern of epigenetic histone posttranslational modifications is regulated by the chromatin kinase VRK1. Loss of VRK1 activity by depletion or pharmacological inhibition causes a change in the pattern of histone PTMs associated with chromatin remodeling. A similar effect can be achieved by the inhibition of KAT and KDM inhibitors. Therefore, the combination of drugs targeting proteins belonging to different types of epigenetic enzymes can be useful for development of novel therapeutic approaches based on synthetic lethality strategies for cancer treatments.

## Methods

### Reagents and inhibitors

The VRK1 inhibitor (VRK-IN-1) [16] was obtained from MedChemExpress (Monmouth Junction, NJ, USA; Cat. No. HY-126542) and used as recommended by the manufacturer.

The inhibitors used in this work are listed in Table 1.

**Table 1:** Inhibitors of histone PTMs

Inhibitor	Target	Concentration/time (24 h)	Supplier	Catalog reference
VRK-IN-1	VRK1	600 nM	MedChem	HY-126542
C646	P300/CBP/PCAF	5 μM	Selleck	S7152
Vorinostat (SAHA)	HDAC1	5 μM	Axon	1785
Entinostat	HDAC1/3	5 μM	Selleck	S1053
Panobinostat	HDAC1	50 nM	Selleck	S1030
Selisistat	HDAC1	50 nM	Selleck	S1541
Chaetocin	KMT1E (SETDB1)	100 nM	Sigma	C9492
Tazemetostat	KMT6A (EZH2)	50 nM	Selleck	S7128
Iadademstat (ORY1001)	KDM1A (LSD1)	50 nM	Selleck	S7795
JMJD2i (Jumonji)	KDM4A (JMJD2A/2E)	100 µM	Millipore	420,201

### Plasmids

The plasmids detailed in Table 2 were used for mammalian cell transfection to express human proteins, or expression in *E. coli* for protein purification. GeneJET Plasmid Maxiprep kit (Thermo Fisher Scientific; Waltham, MA, USA) was used for plasmid purification.

**Table 2:** Plasmids

Protein or tag	Plasmid	Tag	Expression	Supplier, reference
EZH2	pCMV6	Myc-DDK	Mammalian	Origene, #RC202054
Flag-Ø	pCEFL	Flag	Mammalian	Gift from JS Gutkind (NIH)
GST-Ø	pGEX-2TK	GST	Bacterial	GE Healthcare GE28-9546-53
HA-Ø	pCEFL	HA	Mammalian	Gift from JS Gutkind (NIH)
HDAC1	pcDNA3.1+	Flag	Mammalian	Addgene, #13820
KDM1A (LSD1)	pCMV6	Myc-DDK	Mammalian	Origene, #RC208480
KDM3A (JMJD1A)	pLenti	V5-Myc	Mammalian	Addgene, #82331
KDM4A (JMJD2A)	pCMV	HA	Mammalian	Addgene, #24180
PCAF	pCl	Flag	Mammalian	Nakatani [81]
SETDB1	pIRES2-EGFP	Flag	Mammalian	Rodriguez Paredes [82]
VRK1	pCEFL	HA	Mammalian	Valbuena et al. [83]
VRK1	pcDNA3.1	Myc	Mammalian	Valbuena et al. [83]

### Antibodies

The antibodies used in this work and their applications and conditions of primary antibodies are listed in Table 3. Secondary antibodies are in Table 4. Antibodies were diluted in TBS-T buffer (25 mM Tris HCl pH 8.0, 50 mM NaCl and 2.5 mM KCl, 0.1% Tween-20) or PBS-1% BSA for immunoblots or immunofluorescence assays, respectively.

**Table 3:** Primary antibodies

Antibody	Type	Dilution (WB/IF)	Clon, reference	Supplier
H3K4me3	Rabbit polyclonal	1:800	9727	Cell signaling
		1:1000		
H3K9ac	Rabbit polyclonal	1:2000	07-352	Millipore
		1:1000		
H3K9me3	Rabbit polyclonal	1:800	07-442	Millipore
		1:800		
H3K27ac	Rabbit polyclonal	1:1000	07-360	Millipore
		1:1000		
H3K27me3	Rabbit polyclonal	1:800	07-449	Millipore
		1:800		
Histone H3	Rabbit polyclonal	1:1000	9715	Cell signaling
		–		
VRK1	Mouse monoclonal	1:1000	1F6	[83]
		–		
VRK1	Rabbit polyclonal	1:1000	VC	[83]
		–		
HA.11 tag	Mouse monoclonal	1:1000	901514, 16B12	BioLegend
		–		
HA tag	Rabbit polyclonal	1:1000	H6908	Sigma-Aldrich
		–		
Myc tag	Mouse monoclonal	1:1000	4A	Millipore
		–	05-724	
Myc tag	Rabbit polyclonal	1:1000	06-549	Millipore
		–		
V5 tag	Mouse monoclonal	1:1000	V5-10	Sigma-Aldrich
		–	V8012	
V5 tag	Rabbit polyclonal	1:1000	V8137	Sigma-Aldrich
		–		
β-actin	Mouse monoclonal	1:2000	AC15	Sigma-Aldrich
		–	A5441	
Flag tag	Mouse monoclonal	1:1000	M5	Sigma-Aldrich
		–	F4042	
Flag tag	Rabbit polyclonal	1:1000	F7425/ab1162	Sigma-Aldrich/Abcam
		–

**Table 4:** Secondary antibodies

Antibody	Fluorophore	Use dilution	Reference	Supplier
Cy^TM^2-goat anti-rabbit	Cy2 (green)	IF	111-225-144	Jackson Immunoresearch
		1:1000		
Cy^TM^3-goat anti-mouse	Cy3 (red)	IF	15-165-146	Jackson Immunoresearch
		1:1000		
Cy^TM^5-goat anti-mouse	Cy5 (far red)	IF	115-175-146	Jackson Immunoresearch
		1:1000		
Goat anti-mouse IgG	DyLight 680 (red)	WB 1:10,000	35,518	Thermo Fisher Scientific
Goat anti-rabbit IgG	DyLight 800 (green)	WB 1:10,000	35,571	Thermo Fisher Scientific

### Cell lines, culture and transfections

The following cell lines were used and purchased from the American Type Culture Collection (ATCC): A549 (CCL-185), U2OS (HTB-96) and HEK 293T (CRL-3216). Cells were grown with DMEM (Gibco-Life Technologies-Invitrogen; Waltham, MA, USA) containing 10% fetal bovine serum (FBS), 2 mM glutamine (l-glutamine) and 1% penicillin–streptomycin (Pen/Strep), all of them from Gibco-Life Technologies (Waltham, MA, USA). The cell lines were placed in a humidified atmosphere containing 5% CO_2_ at 37 °C. Twenty-four hours later, cells were transfected with the corresponding expression plasmid. Plasmid transfections were performed mixing 4–8 µg DNA with two volumes of polyethylenimine (PEI; Polysciences; Warrington; PA, USA) reagent, incubated for 30 min and added to the cells, which were assayed 48 h after transfection. Serum starvation (DMEM supplemented with 0.5% FBS, 2 mM L-glutamine, 1% Pen/Strep) was performed for 48 h when indicated.

### VRK1 depletion by siRNA

Two specific siRNAs were used for human VRK1 depletion: siVRK1-02 (5′-CAAGGAACCTGGTGTTGAA-3′) and siVRK1-3 (5′-GGAAUGGAAAGUAGGAUUA-3′). ON-TARGET plus siControl non-targeting siRNA (siControl) was used as a negative control. VRK1 depletion was performed as previously reported [50, 79]. All RNAi were from GE-Healthcare-Dharmacon. Both lipotransfectin (Solmeglas, Madrid, Spain) and 200 nM siRNA was diluted in Opti-MEM (GIBCO-Life Technologies) according to manufacturer guidelines. After 30 min of incubation, the mix was added to the cells. Cells were maintained with antibiotic-free medium.

### Cell lysates and histone extraction

Protein extraction was carried out at 4 °C. Cells were resuspended in lysis buffer (50 mM Tris HCl pH 8.0, 150 mM NaCl, 1% triton X-100 and 1 mM EDTA) supplemented by phosphatases inhibitors (1 mM sodium fluoride and 1 mM sodium orthovanadate) and proteases inhibitors (1 mM PMSF, 10 mg/mL aprotinin, and 10 mg/mL leupeptin). The cell lysate suspension was incubated for 15 min and then centrifuged for 15 min (16,000 × g). Histones were isolated by acidic extraction, as previously described [80]. BCA assay (Thermo Fisher Scientific; Waltham, MA, USA) was used to determine the total protein concentration. 40 µg of protein and 5–10 µg of acidic extracts of histones were used for immunoblots.

### Pull-down and immunoprecipitation assays

Pull-down assay was carried out with purified VRK1-His and GST-PCAF (aa 352–832) in the amounts indicated in the experiment. For this aim, purified proteins were incubated in pull-down buffer (20 mM Tris–HCl pH 7.5, 5 mM MgCl2, 0.5 mM DTT and 150 mM KCl) at 37 °C and gentle agitation for 45 min. After that, 40 µL of Glutathione Sepharose 4B beads (GE Healthcare; Chicago, IL, USA) was added, and the mix was incubated overnight at 4 °C in rotation. The pull-down was performed by centrifugation (500 × *g*, 2 min, 4 °C) and the beads were washed in the same buffer for three times. Then, the protein complexes were resuspended in the sample loading buffer, loaded in polyacrylamide gel to separate proteins via SDS-PAGE and visualized by Coomassie Blue staining (3 g/L Coomassie Brilliant Blue R250, 45% methanol, and 10% glacial acetic acid).

The co-immunoprecipitations were performed using 0.5–1 mg of the whole-cells extracts. The protocol consists in incubating the proteins with the specific antibody for 6–8 h at 4 and then adding 40 µL of Protein G–Agarose Resin 4 Rapid Run (4RRPG, Agarose Bead Technologies; Madrid, Spain) overnight at 4 °C in rotation. Finally, the immunoprecipitate was collected by centrifugation (500 × g, 2 min, 4 °C) [30, 31, 38].

### Western blot analysis

Western blot technique was performed according to standardized protocols. Briefly, protein samples were boiled and loaded into a polyacrylamide gel to separate proteins via SDS-PAGE. Then, proteins were transferred to PVDF Immobilon-FL (0.22 or 0.45 µm pore size; Millipore; Burlington, MA, USA) membranes and blocked for 1 h at room temperature with 5% nonfat milk or 5% of BSA in TBS-T buffer. Membranes were incubated with the primary antibody overnight at 4 °C (Table 3). The following day, they were incubated in darkness with their corresponding secondary antibodies (Table 4) diluted 1:10,000 in TBS-T for 1 h. Lastly, fluorescence signals were detected using a LI-COR Odyssey Infrared Imaging System (LI-COR Biosciences; Lincoln, NE, USA). To analyze differences in proteins levels, densitometric analysis was done using ImageJ 1.52p software (https://imagej.nih.gov). All western blots were performed in triplicate and correspond to the accompanying immunofluorescence image.

### Immunofluorescence and confocal microscopy

In this study, cells were cultured with glass coverslips (Thermo Fisher Scientific; Waltham, MA, USA). Cells were fixed with 3% paraformaldehyde (PFA) in PBS for 15 min, and treated with 200 mM glycine solution to remove the PFA. Subsequently, cells were incubated with 0.2% triton X-100 permeabilization for 15 min and then blocked with PBS–1% BSA with 0.1% sodium azide for 1 h at room temperature, or overnight at 4 °C, to block unspecific binding of the antibodies. Cells were consecutively incubated with two primary antibodies for concurrently protein detection. The first primary antibody was incubated between 5 and 6 h at room temperature and next the second primary antibody overnight at 4 °C. Cells were washed in PBS three times for 5 min. Next, cells were incubated with the secondary antibodies at 1:1000 dilution for 1 h at room temperature in the dark. Cells were washed in PBS three times for 5 min in the dark. Nuclei were stained with DAPI (4′,6-diamidino-2-phenylindole) at 1:1000 dilution for 5 min. Finally, cells were washed three times for 5 min in PBS and slides were mounted with Mowiol. Fluorescent images were acquired with a LEICA SP5 DMI-6000B confocal microscope (Leica; Wetzlar, Germany), using the following lasers: Argon (488 nm), DPSS (561 nm) and UV Diode (405 nm). Images were analyzed using the ImageJ software.

### Statistical analysis

GraphPad Prism 8.0 was used to analyze and computed graphs. Experiments were set up with 3 replicates per experiment. Data are presented as dot plots with the median, first and third quartiles and whiskers. Statistical analysis was performed by Mann–Whitney *U*-test test for two-group comparisons after confirming samples did not adjust to a normal distribution (non-parametric distributions) according to two-tailed Kolmogorov test. In all cases, the level of significance was 0.05 (*, *p* < 0.05; **, *p* < 0.01; and ***, *p* < 0.001).

### Supplementary Information

**Additional file 1. Fig. S1**: Effect of VRK1 depletion on the epigenetic modifications of H3K9 in the presence (top) or absence (bottom) of serum in A549 lung adenocarcinoma cells.

**Additional file 2. Fig. S2**: Effect of VRK1 depletion on epigenetic modifications of H3K9 in the presence or absence of serum in U2OS osteosarcoma cells.

**Additional file 3. Fig. S3**: Effect of VRK1 depletion on the epigenetic modifications of H3K27 in the presence or absence of serum in A549 lung adenocarcinoma cells.

**Additional file 4. Fig. S4**: Effect of VRK1 depletion of epigenetic modifications of H3K27 in the presence or absence of serum in U2OS osteosarcoma cells.

**Additional file 5. Fig. S5**: Effect of the VRK-IN-1 inhibitor on the levels of H3K27 acetylation and methylation in A549 cells.

**Additional file 6. Fig. S6**: Effect of the VRK1 depletion and the VRK-IN-1 inhibitor on the levels of H4K16 acetylation and H3K4me3 associated with transcription in U2OS cells.

**Additional file 7. Fig. S7**: Effect of the VRK-IN-1 inhibitor on the levels of H3K9 acetylation and methylation in U2OS cells.

**Additional file 8. Fig. S8**: Effect of the VRK-IN-1 inhibitor on the levels of H3K27 acetylation and methylation in U2OS cells.

**Additional file 9. Fig. S9**: Effect of HDAC inhibitors on H3K9 acetylation and methylation in U2OS cells.

**Additional file 10. Fig. S10**: Effect of KMT inhibitors on H3K9 acetylation and methylation in U2OS cells.

**Additional file 11. Fig. S11**: Effect of the KAT inhibitor C646 on H3K9 acetylation and methylation in A549 and U2OS cells.

**Additional file 12. Fig. S12**: Effect of KDM inhibitors on H3K9 acetylation and methylation in A549 and U2OS cells.

**Additional file 13. Fig. S13**: Direct interaction between VRK1 and the PCAF acetyl transferase.

## Data Availability

All data and reagents are available upon request.
