# Supplementary material for: The pattern of histone H3 epigenetic posttranslational modifications is regulated by the VRK1 chromatin kinase
Source: Epigenetics Chromatin. 2023 May 13;16:18. doi: 10.1186/s13072-023-00494-7 (PMC10182654; doi:10.1186/s13072-023-00494-7)
Supplement: Supplementary file 1 — Additional file 1. Fig. S1: Effect of VRK1 depletion on the epigenetic modifications of H3K9 in the presence (top) or absence (bottom) of serum in A549 lung adenocarcinoma cells. [file 13072_2023_494_MOESM1_ESM.pdf]

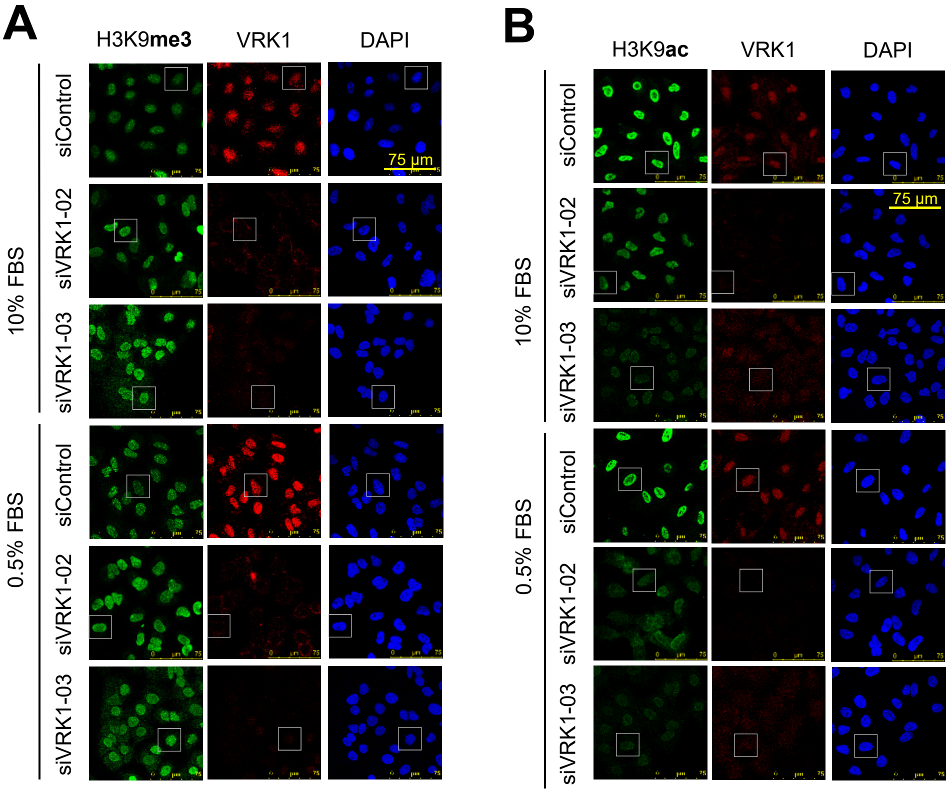

Figure S1. Effect of VRK1 depletion on the epigenetic modifications of H3K9 in the presence (top) or absence (bottom) of serum in A549 lung adenocarcinoma cells. Field images of data shown in Figure 1. The selected cells shown in Figure 1 are indicated by a square. **A.** Effect of VRK1 depletion on histone H3K9 acetylation in A549 cells. **B.** Effect of VRK1 depletion on histone H3K9 trimethylation in A549 cells. The yellow bar indicates size of 75 µm. siCt: siControl; siV-02: siVRK1-02; siV-03: siVRK1-03.
