# Supplementary material for: The pattern of histone H3 epigenetic posttranslational modifications is regulated by the VRK1 chromatin kinase
Source: Epigenetics Chromatin. 2023 May 13;16:18. doi: 10.1186/s13072-023-00494-7 (PMC10182654; doi:10.1186/s13072-023-00494-7)
Supplement: Supplementary file 2 — Additional file 2. Fig. S2: Effect of VRK1 depletion on epigenetic modifications of H3K9 in the presence or absence of serum in U2OS osteosarcoma cells. [file 13072_2023_494_MOESM2_ESM.pdf]

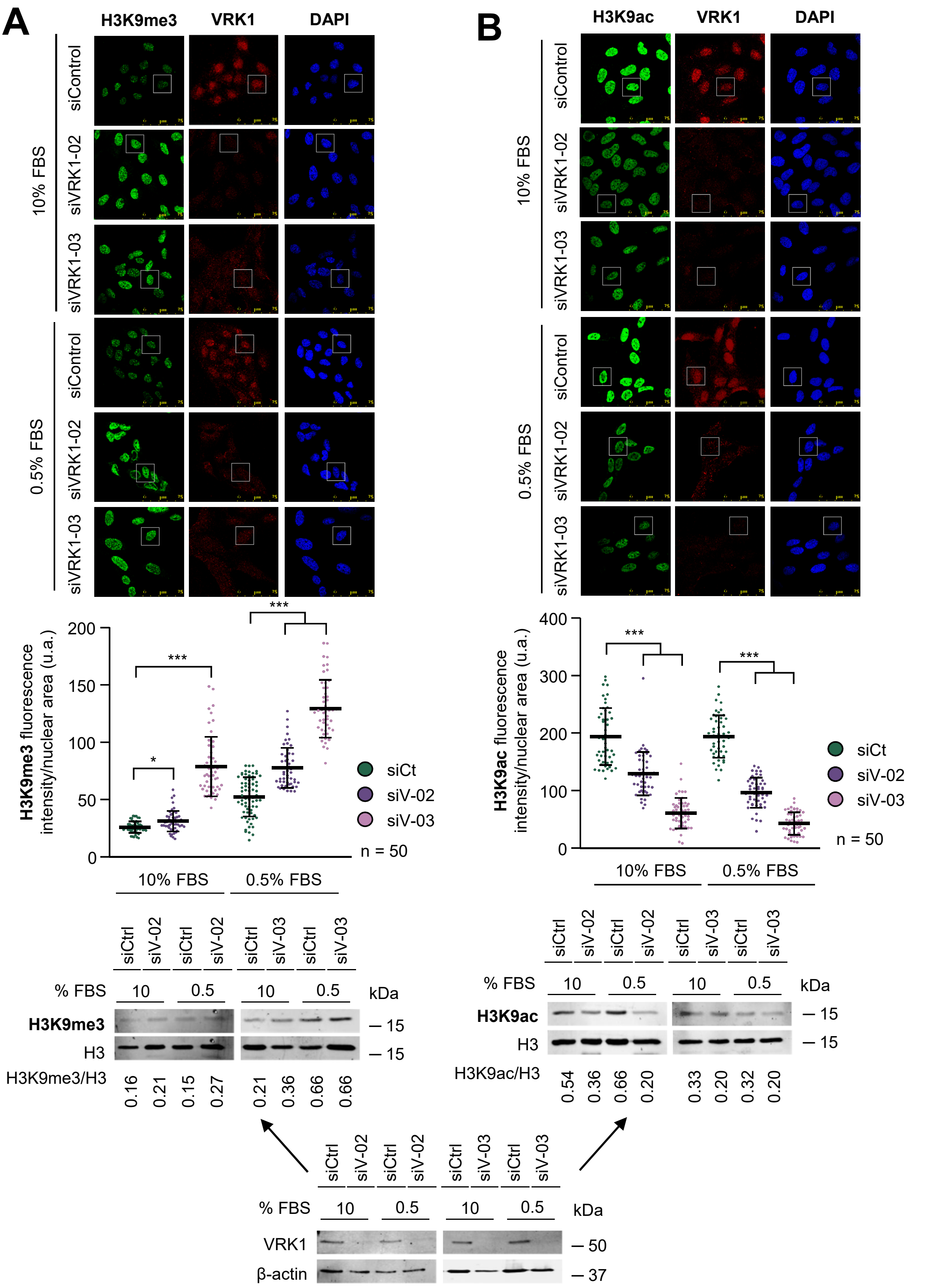

Figure S2. Effect of VRK1 depletion of epigenetic modifications of H3K9 in the presence or absence of serum in U2OS osteosarcoma cells. The field figure is shown in: Figure S2. **A.** Effect of VRK1 depletion on histone H3K9 methylation in U2OS osteosarcoma cells. **B.** Effect of VRK1 depletion on histone H3K9 acetylation in U2OS cells. The quantification of K3K9 methylation (left) and acetylation (right) from 50 cells are shown below. At the bottom are shown the immunoblots of the cell extracts from the experiment. The lysate was divided in two for loading in gels to determine each of the H3K9 modifications. *** $P < 0.001$. siCtrl: siControl; siV-02: siVRK1-02; siV-03: siVRK1-03. The same extract was used for both determinations.
