# Supplementary material for: The pattern of histone H3 epigenetic posttranslational modifications is regulated by the VRK1 chromatin kinase
Source: Epigenetics Chromatin. 2023 May 13;16:18. doi: 10.1186/s13072-023-00494-7 (PMC10182654; doi:10.1186/s13072-023-00494-7)
Supplement: Supplementary file 3 — Additional file 3. Fig. S3: Effect of VRK1 depletion on the epigenetic modifications of H3K27 in the presence or absence of serum in A549 lung adenocarcinoma cells. [file 13072_2023_494_MOESM3_ESM.pdf]

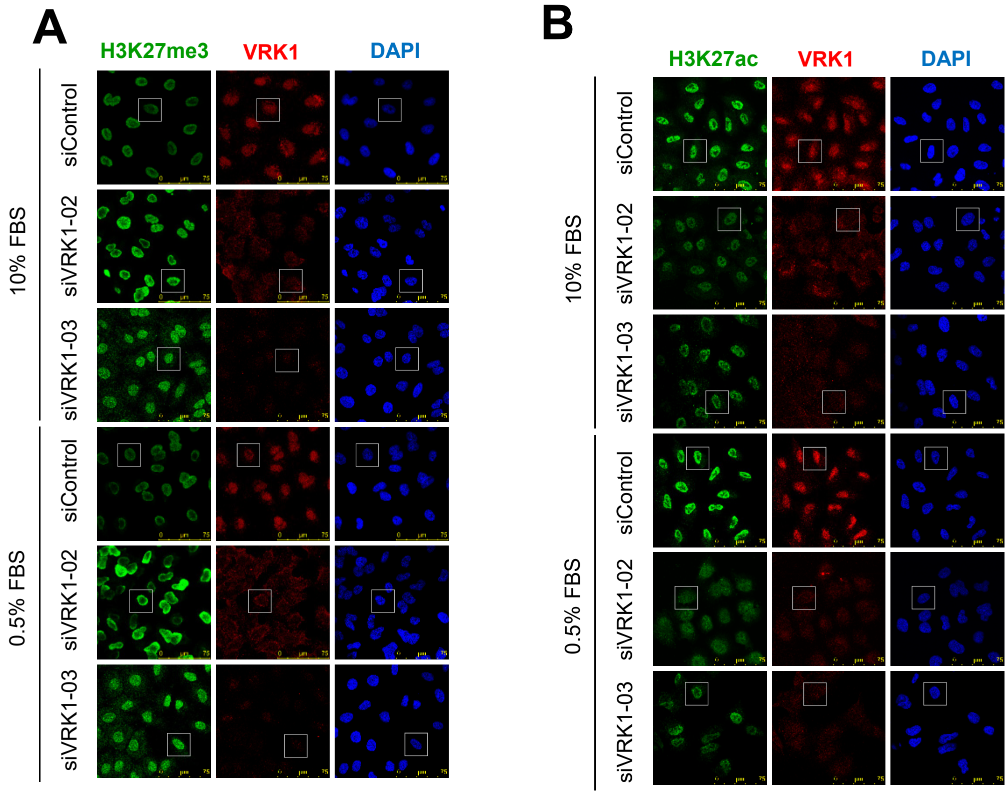

Figure S3. Effect of VRK1 depletion on the epigenetic modifications of H3K27 in the presence (top) or absence (bottom) of serum in A549 lung adenocarcinoma cells. **A.** Effect on H3K27me3. **B.** Effect on H3K27ac. The boxes indicate the selected cells shown in Figure 2. The selected cells are indicated by a square. siCt: siControl; siV-02: siVRK1-02; siV-03: siVRK1-03.
