# Supplementary material for: The pattern of histone H3 epigenetic posttranslational modifications is regulated by the VRK1 chromatin kinase
Source: Epigenetics Chromatin. 2023 May 13;16:18. doi: 10.1186/s13072-023-00494-7 (PMC10182654; doi:10.1186/s13072-023-00494-7)
Supplement: Supplementary file 4 — Additional file 4. Fig. S4: Effect of VRK1 depletion of epigenetic modifications of H3K27 in the presence or absence of serum in U2OS osteosarcoma cells. [file 13072_2023_494_MOESM4_ESM.pdf]

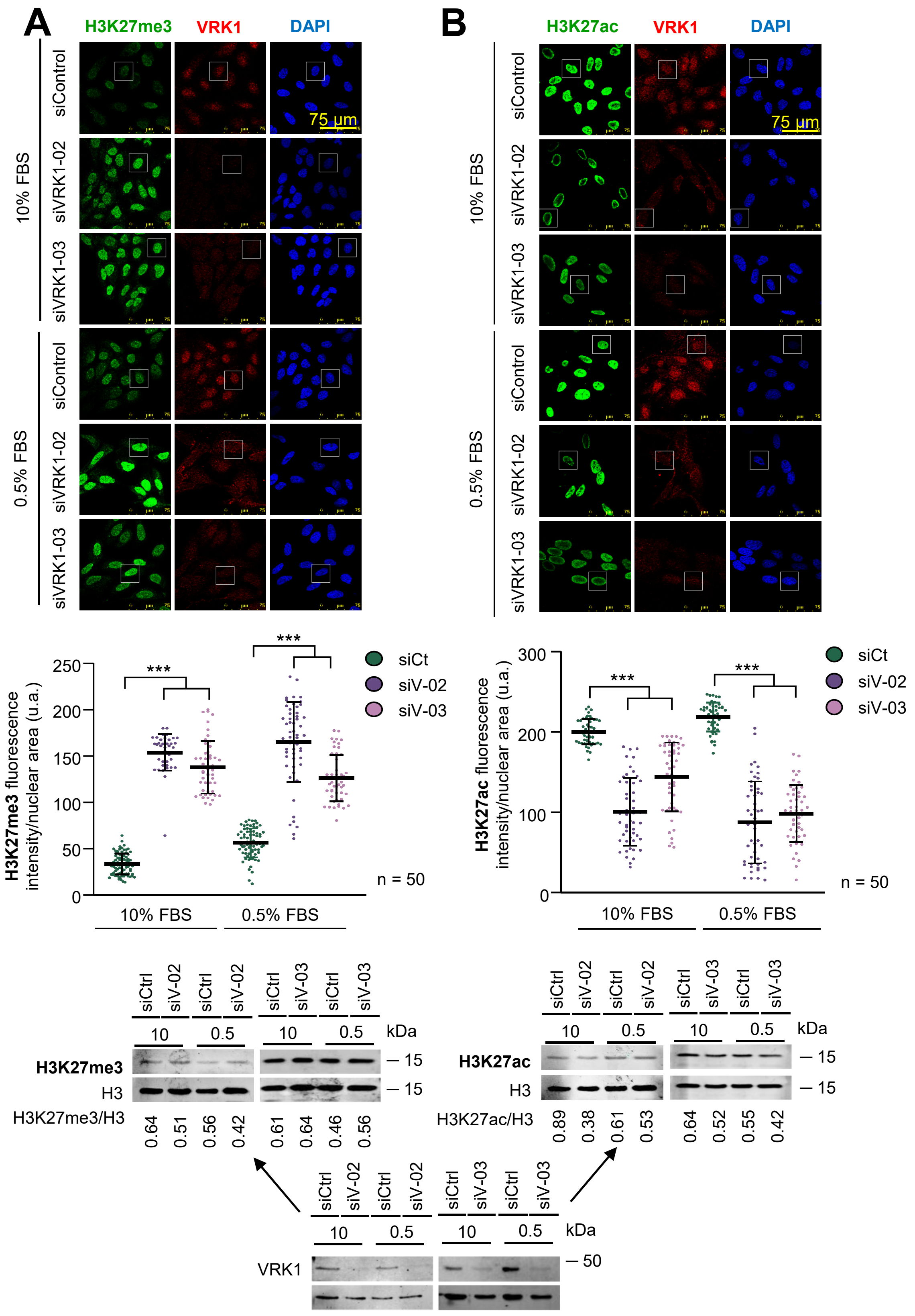

Figure S4. Effect of VRK1 depletion of epigenetic modifications of H3K27 in the presence or absence of serum in U2OS osteosarcoma cells. **A.** Effect of VRK1 depletion on histone H3K27 methylation in U2OS osteosarcoma cells. **B.** Effect of VRK1 depletion on histone H3K27 acetylation in U2OS cells. The quantification of H3K27 methylation (left) and acetylation (right) from 50 cells are shown below. The immunoblots of the cell extracts from the experiment are shown at the bottom. The lysate was divided in two for loading in gels to determine each of the H3K27 modifications. *** $P < 0.001$. siCtrl: siControl; siV-02: siVRK1-02; siV-03: siVRK1-03.
