# Supplementary material for: The pattern of histone H3 epigenetic posttranslational modifications is regulated by the VRK1 chromatin kinase
Source: Epigenetics Chromatin. 2023 May 13;16:18. doi: 10.1186/s13072-023-00494-7 (PMC10182654; doi:10.1186/s13072-023-00494-7)
Supplement: Supplementary file 5 — Additional file 5. Fig. S5: Effect of the VRK-IN-1 inhibitor on the levels of H3K27 acetylation and methylation in A549 cells. [file 13072_2023_494_MOESM5_ESM.pdf]

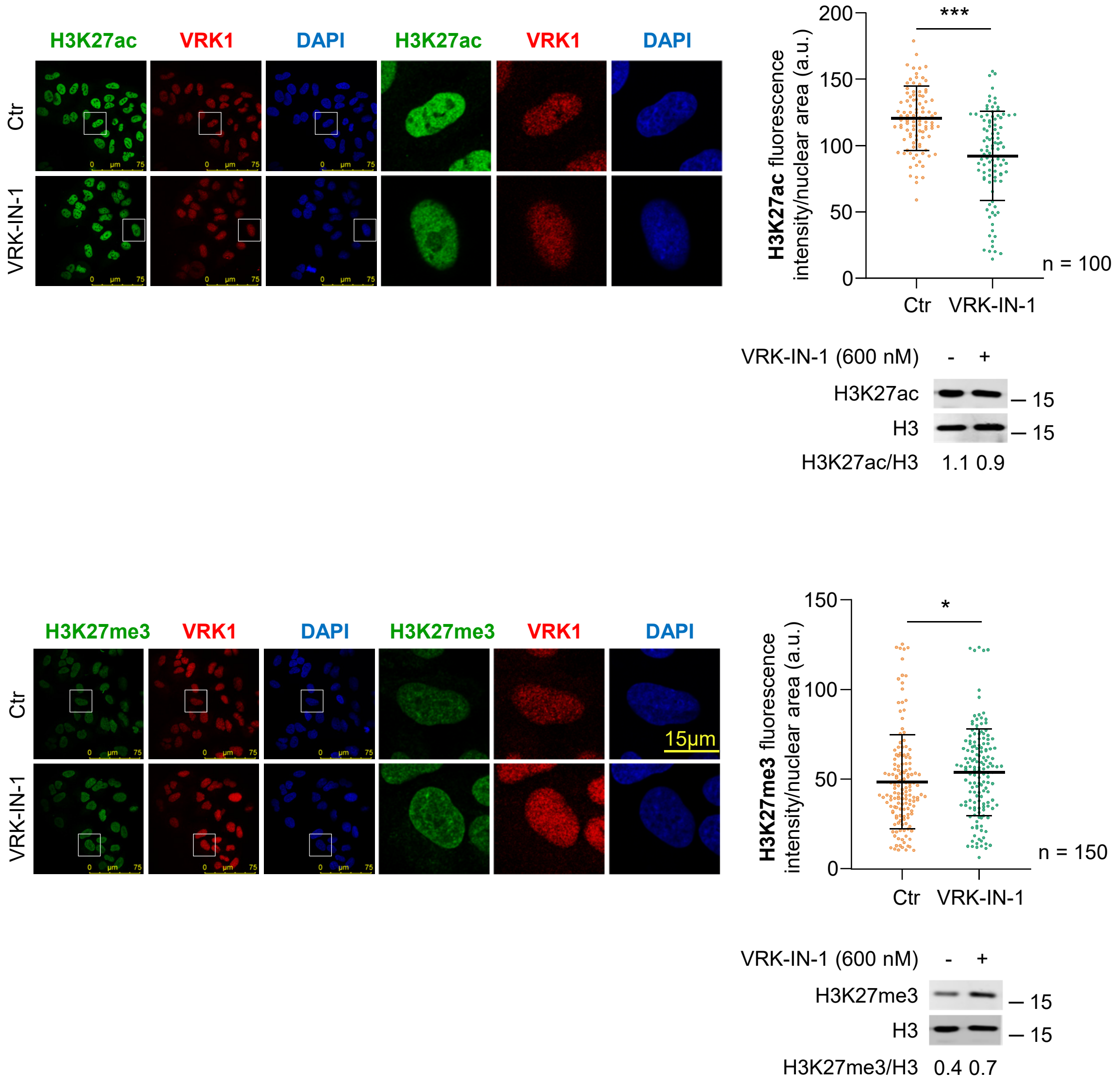

Figure S5. Effect of VRK1 inhibition with VRK1-IN-1 on epigenetic modification of H3K27 acetylation (top) and methylation (bottom) in A549 cells. Ctr: control
