# Supplementary material for: The pattern of histone H3 epigenetic posttranslational modifications is regulated by the VRK1 chromatin kinase
Source: Epigenetics Chromatin. 2023 May 13;16:18. doi: 10.1186/s13072-023-00494-7 (PMC10182654; doi:10.1186/s13072-023-00494-7)
Supplement: Supplementary file 6 — Additional file 6. Fig. S6: Effect of the VRK1 depletion and the VRK-IN-1 inhibitor on the levels of H4K16 acetylation and H3K4me3 associated with transcription in U2OS cells. [file 13072_2023_494_MOESM6_ESM.pdf]

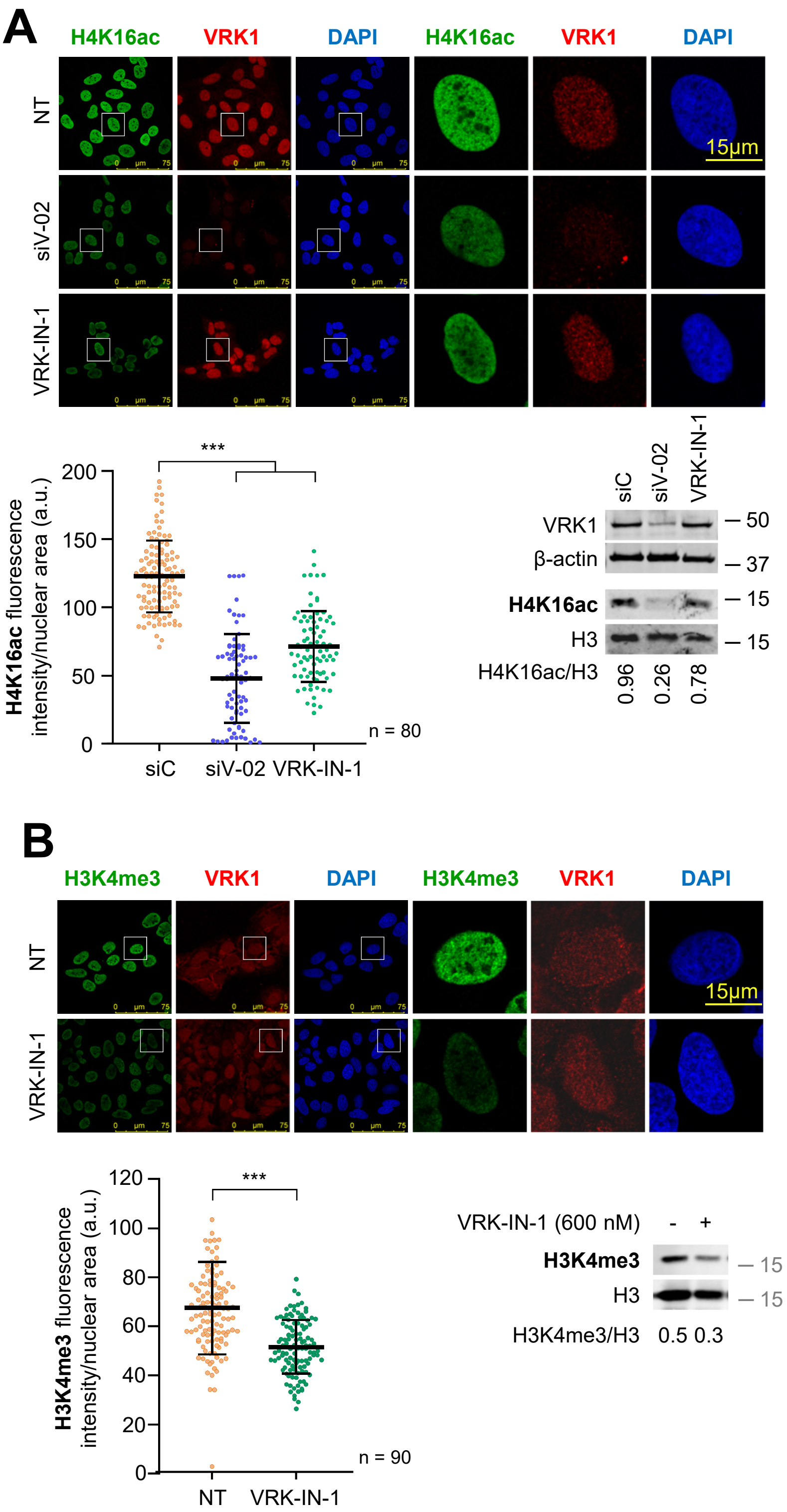

Figure S6. Effect of VRK1 depletion or inhibition with VRK1-IN-1 on epigenetic modifications of H4K16 and H3K4 in U2OS cells. **A.** Effect of depletion and VRK-IN-1 on histone H4K16ac. **B.** Effect of VRK-IN-1 on histone H3K4me3 levels.
