# Supplementary material for: The pattern of histone H3 epigenetic posttranslational modifications is regulated by the VRK1 chromatin kinase
Source: Epigenetics Chromatin. 2023 May 13;16:18. doi: 10.1186/s13072-023-00494-7 (PMC10182654; doi:10.1186/s13072-023-00494-7)
Supplement: Supplementary file 8 — Additional file 8. Fig. S8: Effect of the VRK-IN-1 inhibitor on the levels of H3K27 acetylation and methylation in U2OS cells. [file 13072_2023_494_MOESM8_ESM.pdf]

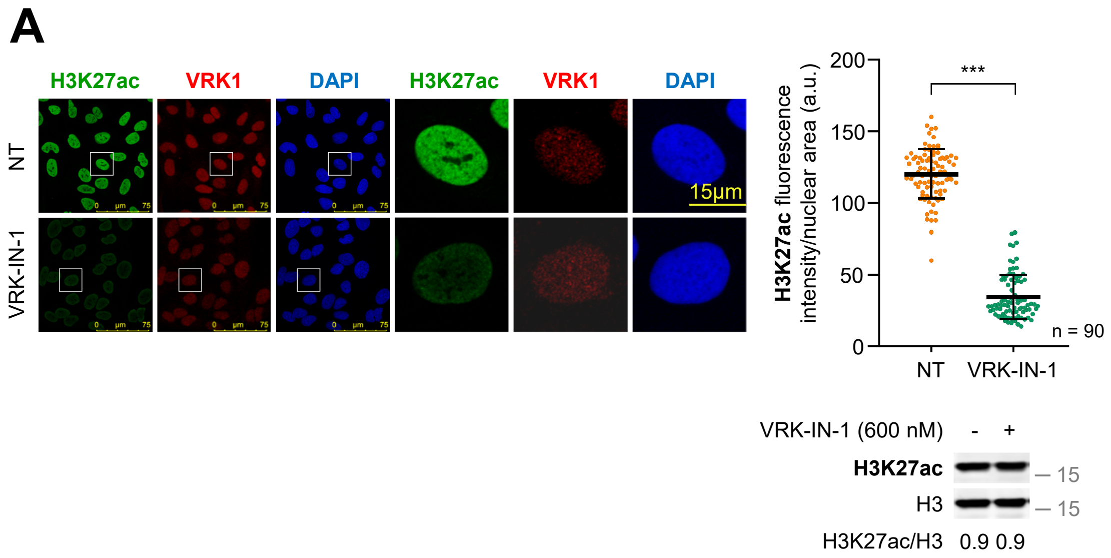

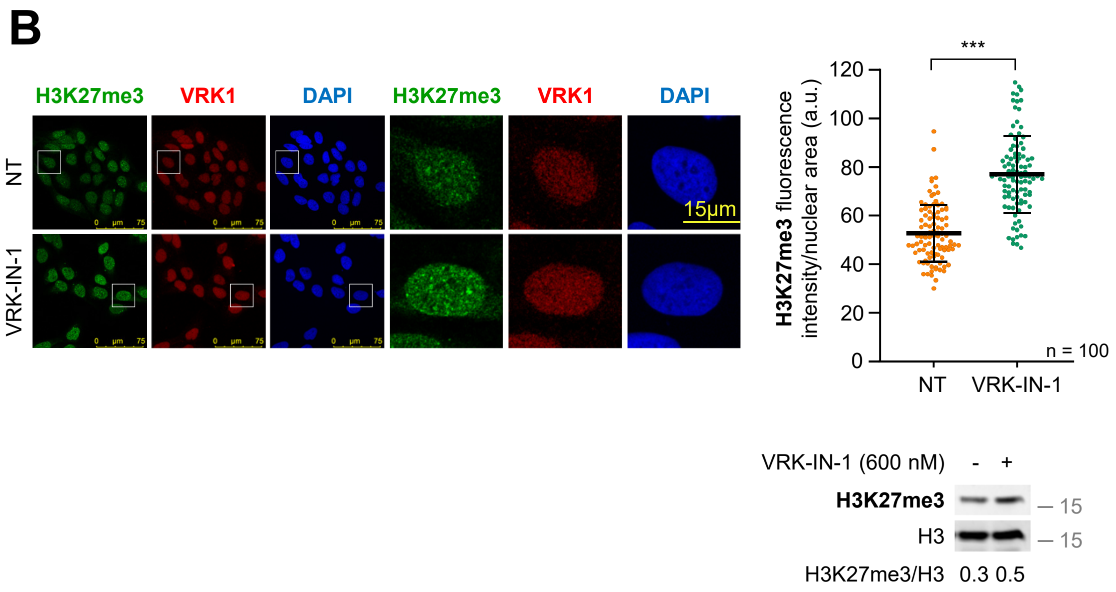

Figure S8. Effect of VRK1 inhibition with VRK1-IN-1 on epigenetic modifications of H3K27 in U2OS cells in the absence of serum. **A**. Effect of VRK-IN-1 on histone H3K27ac levels. **B.** Effect of VRK-IN-1 on histone H3K27m3 levels. NT: not treated.
