# Supplementary figures and images for: The pattern of histone H3 epigenetic posttranslational modifications is regulated by the VRK1 chromatin kinase
Source: Epigenetics Chromatin. 2023 May 13;16:18. doi: 10.1186/s13072-023-00494-7 (PMC10182654; doi:10.1186/s13072-023-00494-7)

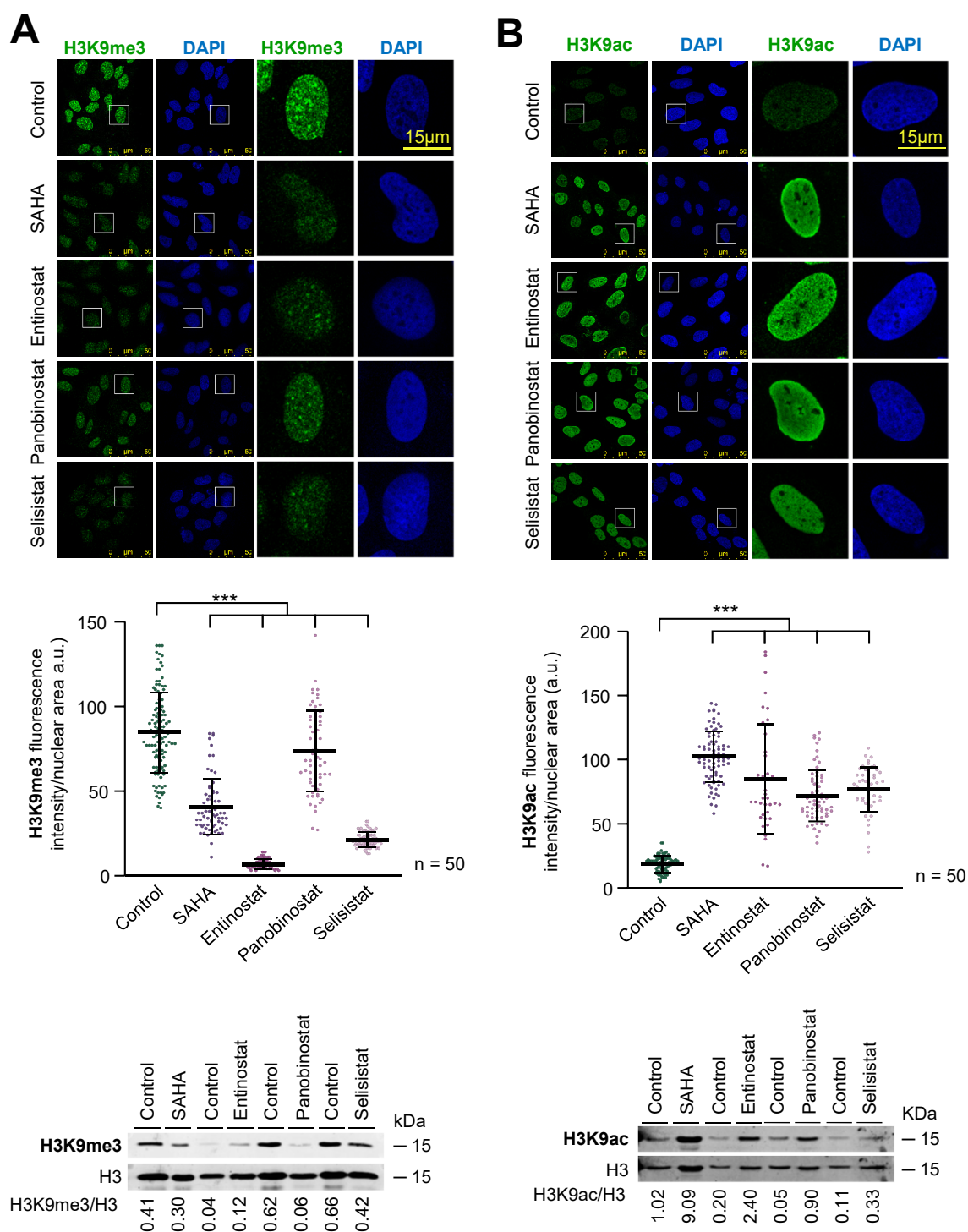

Supplement: Supplementary file 9 — Additional file 9. Fig. S9: Effect of HDAC inhibitors on H3K9 acetylation and methylation in U2OS cells. [file 13072_2023_494_MOESM9_ESM.pdf]
