# Supplementary material for: The pattern of histone H3 epigenetic posttranslational modifications is regulated by the VRK1 chromatin kinase
Source: Epigenetics Chromatin. 2023 May 13;16:18. doi: 10.1186/s13072-023-00494-7 (PMC10182654; doi:10.1186/s13072-023-00494-7)
Supplement: Supplementary file 10 — Additional file 10. Fig. S10: Effect of KMT inhibitors on H3K9 acetylation and methylation in U2OS cells. [file 13072_2023_494_MOESM10_ESM.pdf]

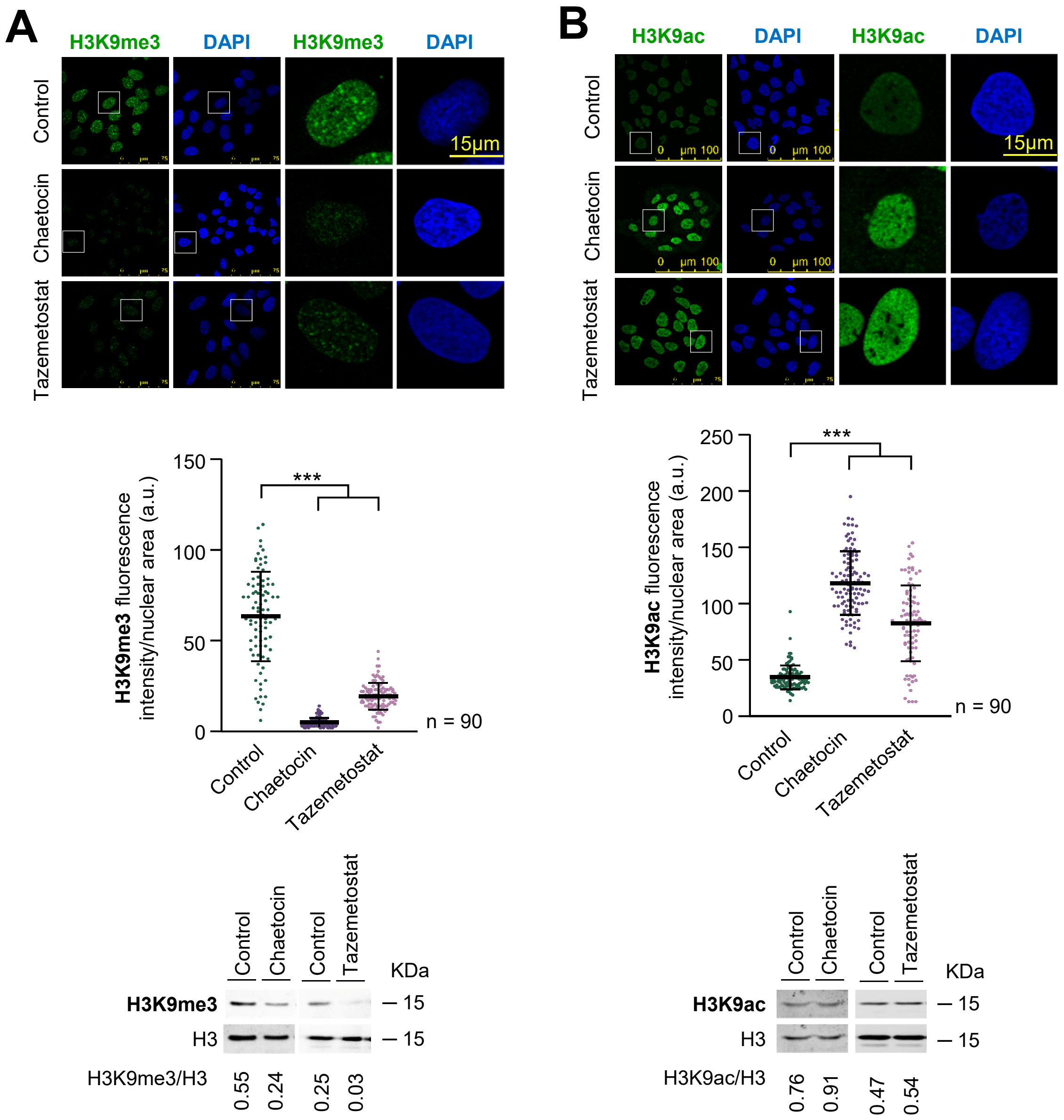

Figure S10. Effect of KMT inhibitors, tazemetostat and chaetocin, on histone H3K9 epigenetic modifications in serum-deprived U2OS cells. **A.** Effect of KMT inhibitors on H3K9m3 levels. **B.** Effect of KMT inhibitors on H3K9ac levels. The middle panel shows the quantification of the fluorescence. The immunoblots of the two modifications are shown at the bottom.
