# Supplementary material for: The pattern of histone H3 epigenetic posttranslational modifications is regulated by the VRK1 chromatin kinase
Source: Epigenetics Chromatin. 2023 May 13;16:18. doi: 10.1186/s13072-023-00494-7 (PMC10182654; doi:10.1186/s13072-023-00494-7)
Supplement: Supplementary file 11 — Additional file 11. Fig. S11: Effect of the KAT inhibitor C646 on H3K9 acetylation and methylation in A549 and U2OS cells. [file 13072_2023_494_MOESM11_ESM.pdf]

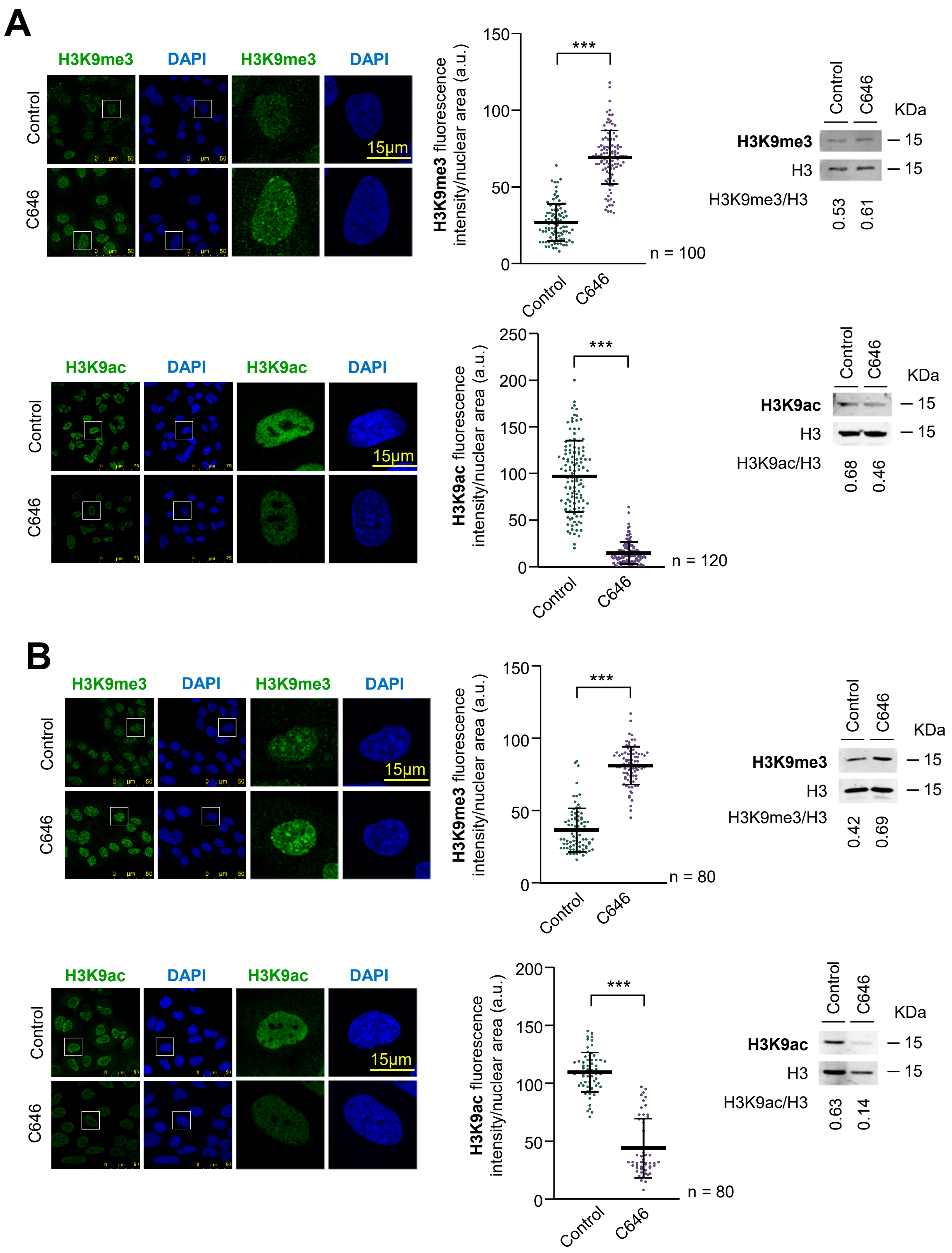

Figure S11. Effect of KAT inhibitor C646 on H3K9 acetylation and methylation in A549 (**A**) and U2OS (**B**) cells. The quantification of the fluorescence is shown in the middle. The immunoblots are shown on the right.
