# Supplementary material for: The pattern of histone H3 epigenetic posttranslational modifications is regulated by the VRK1 chromatin kinase
Source: Epigenetics Chromatin. 2023 May 13;16:18. doi: 10.1186/s13072-023-00494-7 (PMC10182654; doi:10.1186/s13072-023-00494-7)
Supplement: Supplementary file 12 — Additional file 12. Fig. S12: Effect of KDM inhibitors on H3K9 acetylation and methylation in A549 and U2OS cells. [file 13072_2023_494_MOESM12_ESM.pdf]

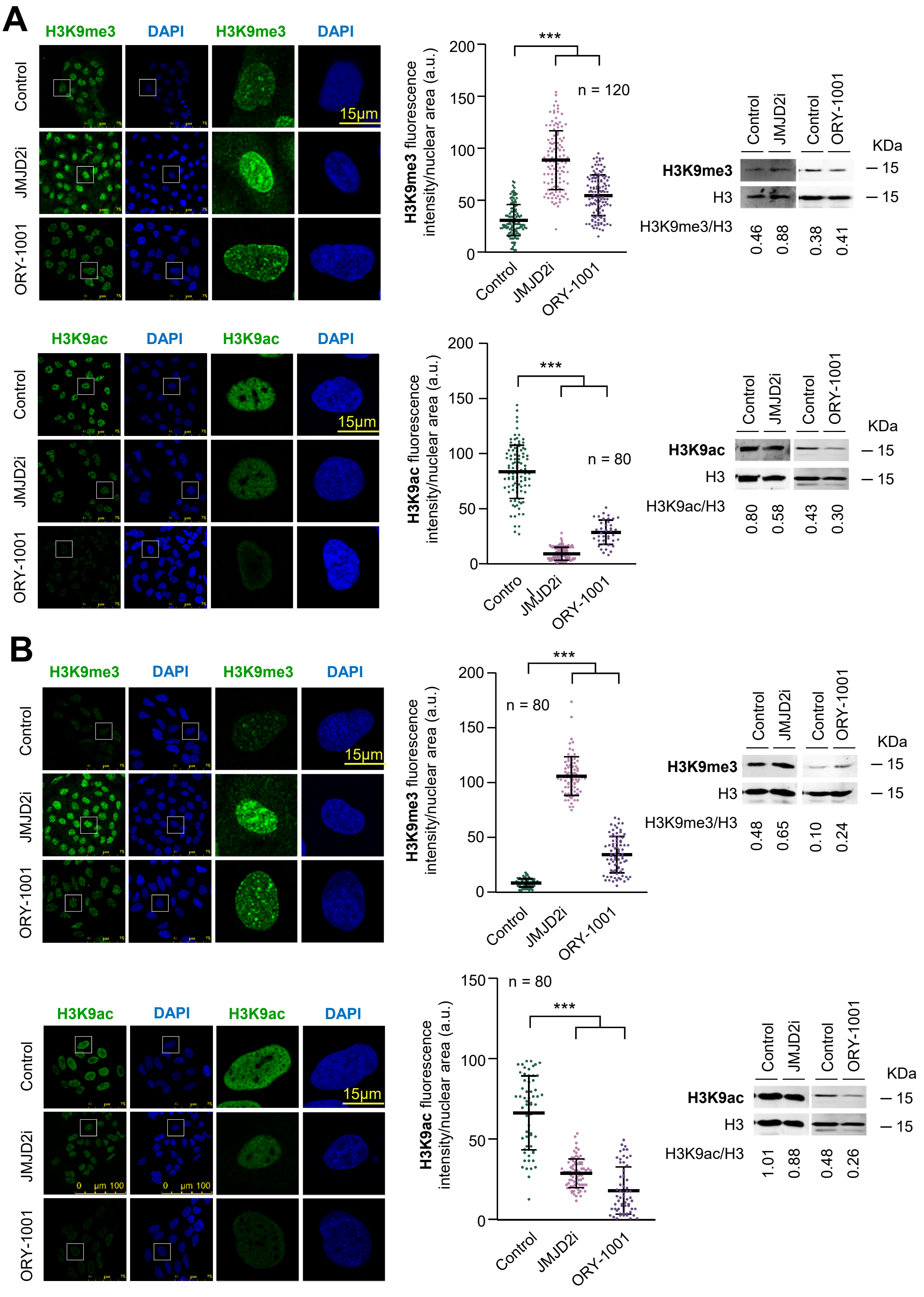

Figure S12. Effect of KDM inhibitors on H3K9 acetylation and methylation in A549 (**A**) and U2OS (**B**) cells. The quantification of the fluorescence is shown in the middle. The immunoblots are shown on the right.
