# Supplementary material for: The pattern of histone H3 epigenetic posttranslational modifications is regulated by the VRK1 chromatin kinase
Source: Epigenetics Chromatin. 2023 May 13;16:18. doi: 10.1186/s13072-023-00494-7 (PMC10182654; doi:10.1186/s13072-023-00494-7)
Supplement: Supplementary file 13 — Additional file 13. Fig. S13: Direct interaction between VRK1 and the PCAF acetyl transferase. [file 13072_2023_494_MOESM13_ESM.pdf]

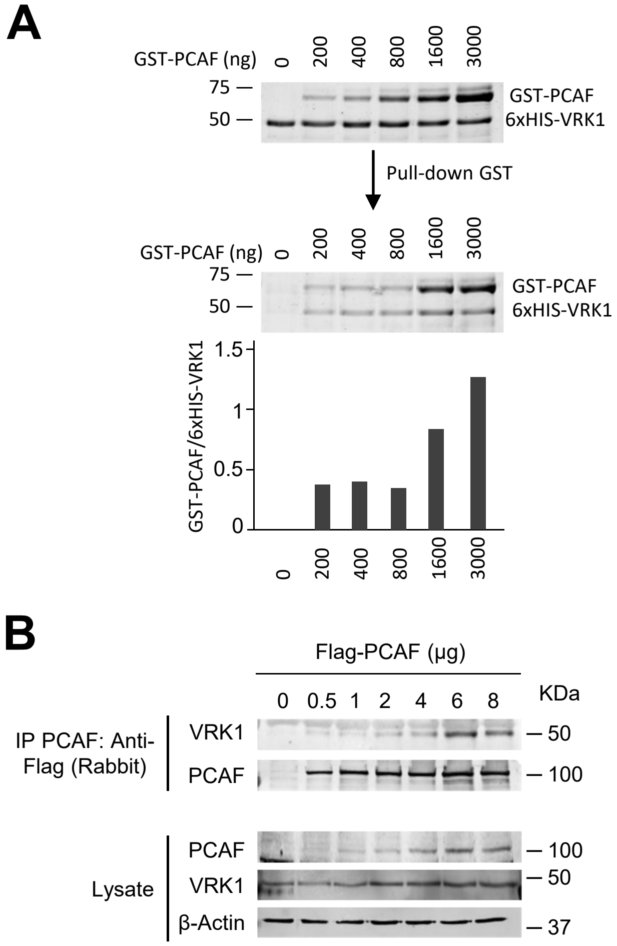

Figure S13. Dose-dependent interaction between VRK1 and the PCAF acetyl transferase. **A**. In vitro pulldown assay with increasing amounts of purified GST-PCAF and his-VRK1 (1 µg. **B.** Interaction of endogenous VRK1 with increasing amounts of PCAF-Flag transfected in HEK293T cells.
